# Estimation of the dispersal distances of an aphid-borne virus in a patchy landscape

**DOI:** 10.1371/journal.pcbi.1006085

**Published:** 2018-04-30

**Authors:** David R. J. Pleydell, Samuel Soubeyrand, Sylvie Dallot, Gérard Labonne, Joël Chadœuf, Emmanuel Jacquot, Gaël Thébaud

**Affiliations:** 1 BGPI, INRA, Montpellier SupAgro, Univ. Montpellier, Cirad, TA A-54/K, Campus de Baillarguet, 34398, Montpellier cedex 5, France; 2 ASTRE, INRA, CIRAD, Univ. Montpellier, Montpellier, France; 3 BioSP, INRA, 84914, Avignon, France; CNRS, FRANCE

## Abstract

Characterising the spatio-temporal dynamics of pathogens *in natura* is key to ensuring their efficient prevention and control. However, it is notoriously difficult to estimate dispersal parameters at scales that are relevant to real epidemics. Epidemiological surveys can provide informative data, but parameter estimation can be hampered when the timing of the epidemiological events is uncertain, and in the presence of interactions between disease spread, surveillance, and control. Further complications arise from imperfect detection of disease and from the huge number of data on individual hosts arising from landscape-level surveys. Here, we present a Bayesian framework that overcomes these barriers by integrating over associated uncertainties in a model explicitly combining the processes of disease dispersal, surveillance and control. Using a novel computationally efficient approach to account for patch geometry, we demonstrate that disease dispersal distances can be estimated accurately in a patchy (i.e. fragmented) landscape when disease control is ongoing. Applying this model to data for an aphid-borne virus (*Plum pox virus*) surveyed for 15 years in 605 orchards, we obtain the first estimate of the distribution of flight distances of infectious aphids at the landscape scale. About 50% of aphid flights terminate beyond 90 m, which implies that most infectious aphids leaving a tree land outside the bounds of a 1-ha orchard. Moreover, long-distance flights are not rare–10% of flights exceed 1 km. By their impact on our quantitative understanding of winged aphid dispersal, these results can inform the design of management strategies for plant viruses, which are mainly aphid-borne.

## Author summary

In spatial epidemiology, dispersal kernels quantify how the probability of pathogen dissemination varies with distance from an infection source. Spatial models of pathogen spread are sensitive to kernel parameters; yet these parameters have rarely been estimated using field data gathered at relevant scales. Robust estimation is rendered difficult by practical constraints limiting the number of surveyed individuals, and uncertainties concerning their disease status. Here, we present a framework that overcomes these barriers to permit inference for a between-patch transmission model. Extensive simulations show that dispersal kernels can be estimated from epidemiological surveillance data. When applied to such data collected from more than 600 orchards during 15 years of a plant virus epidemic our approach enables the estimation of the dispersal kernel of infectious winged aphids. This kernel is long-tailed, as 50% of infectious aphids leaving a tree terminate their infectious flight beyond 90 m whilst 10% fly beyond 1 km. This first estimate of flight distances at the landscape scale for aphids–a group of vectors transmitting numerous viruses–is crucial for the science-based design of control strategies targeting plant virus epidemics.

## Introduction

Infectious diseases of humans, animals and plants severely impact the world’s health and economy. To gain knowledge on disease dynamics, powerful mathematical models have been developed [1–3]. However, for predicting the relative efficacies of competing control strategies across realistic heterogeneous landscapes, spatially-explicit *in silico* simulation models provide the main avenue [2]. The dispersal parameters of such models critically affect the predicted spatio-temporal dynamics of the disease, and thus the predicted outcome of potential control strategies [4]. Obtaining reliable estimates for these parameters is therefore a fundamental issue in epidemiology [5–7]. Models frequently employ dispersal kernels to represent how the probability of dispersal events diminishes as a function of distance, and simulation studies have proven that dispersal parameters can be identified in idealised scenarios [5]. Indeed, this has been achieved for simple models or small-scale datasets [8–13]. Recent advances in Bayesian methods and computing power have enabled fitting more realistic models to larger-scale surveillance data [6, 14–19]. However, most dispersal kernels are still unknown. Indeed, estimation gets more complex when graduating from idealised toy problems to reconstructing the spatio-temporal dynamics of real epidemics. The first issue is the mismatch between the spatio-temporal coordinates of the epidemic, sampling and model [20]. For example, the timing of key events (e.g. when a susceptible individual becomes infected) is often censored (i.e. known only within certain bounds), and failure to account for this can bias estimates. Moreover, the challenge of inference is increased by uncertainty arising from missing observations [21, 22] or imperfect sensitivity of disease detection [23, 24]. Further difficulties arise when surveillance data are aggregated at the patch scale because a landscape comprising patches of various shapes or sizes often cannot be summarized by patch centroids without biasing connectivity estimates. All these issues require appropriate correction measures to avoid biased inference and prediction [25].

In the case of aerial vector- or wind-borne diseases, dispersal kernels critically depend on the flight properties of the vectors or infectious propagules [26]. When the probability of dispersal decreases more slowly than an exponential distribution, kernels are termed “long-tailed” and lead to non-negligible long-distance flights [27]. Such events are an important component of disease epidemiological–and evolutionary–dynamics and call for kernel estimation at the landscape scale [28]. However, among plant diseases, there are few available kernel estimates. The dispersal kernel of black Sigatoka (a fungal disease of banana) has been estimated experimentally up to 1 km from a point source, based on the direct observation of spore-induced lesions [29]. This is the only available direct estimate at this scale for the dispersal kernel of a plant disease, which reflects the extreme practical difficulties of such field studies and highlights the critical need for developing *in silico* solutions. A promising way forward is to infer dispersal parameters indirectly, i.e. from spatio-temporal patterns observed in epidemiological data [5] whilst accounting for the added complexity (outlined above) of observational studies. This approach has been used to infer the dispersal kernels of the wind-dispersed plantain fungus *Podosphaera plantaginis* [15], the fungus *Leptosphaeria maculans* affecting oilseed rape and dispersed both by wind and wind-driven rain [30], and two pathogens transmitted only by wind-driven rain: the oomycete *Phytophthora ramorum* that is responsible for sudden oak death [16], and the bacterium *Xanthomonas axonopodis* that causes Citrus canker [17]. A dispersal kernel has been estimated for two other Citrus diseases: Bahia bark scaling of Citrus, a disease with an elusive etiology [13], and Huanglongbing, which is caused by bacteria from the ‘*Candidatus* Liberibacter’ genus and transmitted by psyllids [18]. To date, this is the only vector-borne plant disease for which the dispersal kernel is documented. Although aphids are responsible for transmitting almost 40% of more than 700 plant viruses [31] and impose large economic burdens, their dispersal remains ill-characterized at the landscape scale [32, 33]. For a vast number of aphid-borne diseases, this lack of basic knowledge affects science-based control strategies by undermining the reliability of quantitative risk assessment and predictive epidemiological models.

Most aphid-borne viruses belong to the *Potyvirus* genus and are transmitted in a non-persistent manner, i.e. by winged aphids that acquire and transmit the virus immediately while probing on various plants in search of a suitable host species [31]. Potyviruses are transmitted by a wide range of aphid species, and aphid infectivity is lost after the first probes. For these reasons, estimating the natural dispersal kernel of a potyvirus provides an indirect way of estimating the dispersal kernel of infectious winged aphids. *Plum pox virus* (PPV) is a potyvirus that is listed as one of the 10 most important plant viruses [34]. This virus is the causal agent of sharka, a quarantine disease affecting trees of the *Prunus* genus (i.e. mainly peach, apricot and plum), reducing fruit yield, quality (modified sugar content and texture) and visual appeal (due to deformations and discolouration) [33]. Sharka is a worldwide plague that has infected over 50 countries in Europe, Asia, America and Africa [33], inflicting estimated economic losses of 10 billion Euros over 30 years [35]. The transfer of infected (possibly symptomless) plant material can disseminate PPV over long distances [35], and the natural spread of the disease is ensured by more than 20 aphid species [36]. Virus-infected trees cannot be cured, and insecticides do not act fast enough to prevent the spread of the virus by non-colonising aphids [31, 37]. In addition, resistant or tolerant peach and apricot varieties are too scarce to provide a short-term alternative to cultivated varieties. However, aphid-mediated transmission can be reduced by removing infected trees as soon as they are detected. As a result, various countries have implemented PPV eradication or control strategies based on regular surveys and removal of trees or orchards when PPV is detected [33, 35, 38]. Given the cost of surveillance, tree removal and compensation, these strategies should benefit from model-assisted optimisation, which requires estimating the aphid dispersal kernel.

In this context, the aims of this study are: (i) to develop a Bayesian inference framework for estimating, from surveillance data, the parameters of a spatially-explicit epidemiological model that accounts for patch geometry and for interactions between disease spread, surveillance and control, (ii) to assess through simulations the accuracy and precision of the dispersal parameters estimated under various epidemic scenarios, and (iii) to apply our method to 15 years of geo-referenced surveillance data collected during an epidemic of *Plum pox virus* in order to estimate the dispersal kernel of the aphid vectors.

## Materials and methods

### Surveillance database

In the early 1990’s, an outbreak of the M strain of PPV was detected in peach/nectarine patches (orchards) in southern France [39]. The plant health services implemented a control strategy based on disease surveillance and removal of symptomatic trees. This process involved the routine collection of patch-level data comprising the observed number of new cases (trees with PPV-typical discolouration symptoms on flowers and leaves) and the corresponding inspection dates, as well as patch attributes (location, planting and removal years, planting density, etc.). We aggregated the information about a 5.6×4.8 km production area over surveillance years 1992-2006 into a unique georeferenced database, with patch boundary coordinates obtained from digitised aerial photographs. With 4820 inspections over 15 years in 553 patches (mean area: 0.95 ha; 52 orchards were replanted in these patches during that period), this database is a precious resource for inference on aphid-mediated viral dispersal in patchy (i.e. fragmented) landscapes. Moreover, to account for seasonal variation in the number of flying aphids, we used in our model the average (over 17 years) weekly number of flying aphids collected from a 12-m-high Agraphid suction tower located within the bio-geographical region of the study area.

### Modelling framework

**Fig 1:**
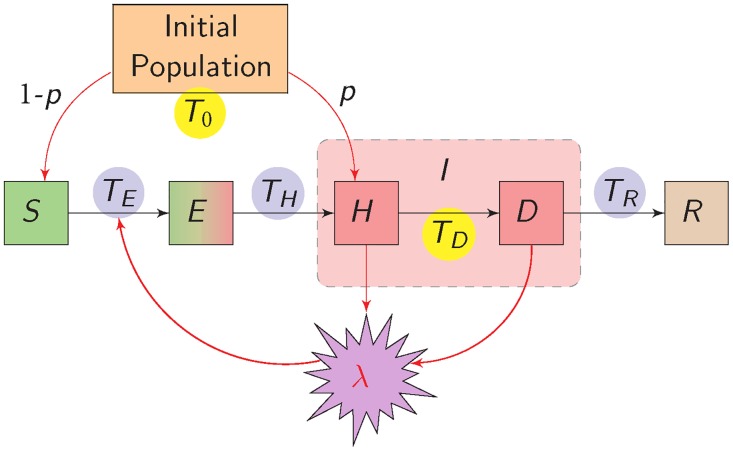
Susceptible-Exposed-Hidden-Detected-Removed (*SEHDR*) model of an individual’s epidemiological status. At *T*_0_, patch *i* is planted with infectious (*I*) or susceptible (*S*) individuals with probabilities *p*_*i*_ and 1-*p*_*i*_, respectively. An individual passes between compartments at event times *T*_*E*_, *T*_*H*_, *T*_*D*_ and *T*_*R*_. Apart from *T*_0_, only the detection time *T*_*D*_ can be known (yellow); all other event times are censored (blue). Infectious individuals from both within and outside the patch contribute to the force of infection $\lambda_{t_{r}}$, which is the expected number of infectious events affecting an individual over time interval (*t*_*r*−1_, *t*_*r*_]. The probability that a given susceptible (*S*) individual becomes exposed (*E*) in this time interval is 1-exp(-$\lambda_{t_{r}}$), assuming independent infection events. A latent period of duration *T*_*H*_-*T*_*E*_ follows, after which the individual becomes infectious (*H*). Infectious individuals are removed (*R*) only after detection (*D*) or when the entire patch is removed. For simplicity, the *i* and *t*_*r*_ subscripts are omitted in the figure.

Our model has a compartmental Susceptible-Exposed-Infectious-Removed (*SEIR*) structure that aims to reduce bias in parameter estimates by accounting for irregular patch geometry, detection-dependent removal, imperfect detection sensitivity, interval censoring of between-compartment transition times, missing data and parameter uncertainty. We address these challenges by: (i) integrating a mixture of exponential dispersal kernels over source and receiver patches to compute between-patch connectivity; (ii) splitting the infectious state *I* into hidden (*H*) and detected (*D*) sub-states (Fig 1); (iii) integrating over uncertainty in the times of transition between compartments; (iv) using Bayesian data augmentation and inference. Two versions of our discrete-time spatio-temporal *SEHDR* model–one for stochastic simulations and the other for Bayesian inference–are described below (for further details, see Texts A and B in S1 Texts).

### Simulation model

Whole patches are removed and replanted at predefined dates throughout the study period. Each patch *i* is planted with *N*_*i*_ individuals. At the planting date, a proportion *p*_*i*_ of these individuals are infectious (in state *H*) and 1-*p*_*i*_ are susceptible (in state *S*). If patch *i* is an introduction patch, *p*_*i*_>0; otherwise, *p*_*i*_ = 0. Up to four transition times (*T*_*E*_, *T*_*H*_, *T*_*D*_ and *T*_*R*_) can be associated with any given individual (Fig 1), i.e. individuals pass sequentially from state *S* to *E* to *H* to *D* to *R*, and all other transitions occur with zero probability. The exposed state *E* accounts for the latent period, i.e. the time-lag between the infection date *T*_*E*_ and the date at which the individual becomes infectious *T*_*H*_. In this discrete-time model (whose time steps are denoted by the index *r*), the transitions (denoted by ‘→’) between the five compartments are modelled as:

$${\vec{SE}}_{i,t_{r}}∼\text{Binom}(S_{i,t_{r-1}},1-e^{-\lambda_{i,t_{r}}}),$$ (1)

$$\text{lag}(\vec{EH})∼{\text{Gamma}}_{\text{Tr}}\left(\theta_{1},\theta_{2}\right),$$ (2)

$${\vec{HD}}_{i,t_{r}}∼\text{Binom}(H_{i,t_{r-1}},\rho_{i,t_{r}}),$$ (3)

$$\text{lag}(\vec{DR})∼{\text{Geom}}_{\text{Tr}}\left(1/\delta \right),$$ (4)

where: $S_{i,t_{r-1}}$ (resp. $H_{i,t_{r-1}}$) is the number of individuals in patch *i* that are in state *S* (resp. *H*) at the beginning of the time interval (*t*_*r*−1_, *t*_*r*_], and ${\vec{SE}}_{i,t_{r}}$ (resp. ${\vec{HD}}_{i,t_{r}}$) represents how many of them make the transition from *S* to *E* (resp. from *H* to *D*) in this time interval; the corresponding transition probabilities are $1-e^{-\lambda_{i,t_{r}}}$ for a given individual in state *S* to incur at least one infection event (transmission of non-persistent viruses is principally driven by independent vectors), and $\rho_{i,t_{r}}$ for the detection of symptoms on an infectious (*H*) individual ($\rho_{i,t_{r}}=\rho$ when patch *i* is inspected in (*t*_*r*−1_, *t*_*r*_], and $\rho_{i,t_{r}}=0$ otherwise); the sojourn times in compartments *E* and *D* are determined per individual via random variables $\text{lag}(\vec{EH})=T_{H}\text{-}T_{E}$ and $\text{lag}(\vec{DR})=T_{R}\text{-}T_{D}$, respectively; the latent period is modelled classically with the flexible gamma distribution, and here the left truncation of Gamma_Tr_ represents an absolute minimal latent period for sharka [33] to account for seasonality in *Prunus* phenology and prevent secondary transmission prior to the first winter; the delay between detection and removal is modelled with a geometric distribution where the probability of removal is the same (1/*δ*) at each time step, up to the right truncation of Geom_Tr_ which represents the maximal delay before removal (detected trees must be removed before the end of the year). The force of infection (i.e. the expected number of transmission events) incurred by each individual in patch *i* over (*t*_*r*−1_, *t*_*r*_] is defined as:

$$\begin{array}{c} \lambda_{i,t_{r}}=\frac{\alpha_{t_{r}}\beta }{N_{i}-R_{i,t_{r-1}}}\sum_{i^{\prime }}(m_{i^{\prime }i}I_{i^{\prime },t_{r-1}}), \end{array}$$ (5)

where $\alpha_{t_{r}}$ is the normalized flight density, i.e. the proportion of annual flights occurring over (*t*_*r*−1_, *t*_*r*_]; *β* is the transmission coefficient, i.e. the annual number of vector flights per source (infectious) host that would lead to infection if the recipient host is susceptible; $N_{i}-R_{i,t_{r-1}}$ is the number of remaining hosts on which the incoming vectors distribute themselves in patch *i*, and $I_{i^{\prime },t_{r-1}}$ is the number of infectious hosts in patch *i*′ over (*t*_*r*−1_, *t*_*r*_]. Note that *N*_*i*_ is constant (i.e. $N_{i}=S_{i,t_{r}}+E_{i,t_{r}}+I_{i,t_{r}}+R_{i,t_{r}}$) for all *t*_*r*_ between the planting and removal dates of patch *i*. Finally, the connectivity *m*_*i*′*i*_ is the probability that a vector flight starting in patch *i*′ terminates in patch *i*.

The connectivity between source patch *i*′ of area $A_{i^{\prime }}$ and receiver patch *i* is obtained via:

$$\begin{array}{c} m_{i^{\prime }i}=\frac{\int_{x\in i^{\prime }}\int_{y\in i}f^{2D}(\|x-y\|)dydx}{A_{i^{\prime }}}, \end{array}$$ (6)

where ***x*** and ***y*** are coordinate vectors in ℝ^2^, and *f*^2*D*^ is the 2-dimensional dispersal kernel [40]. The computation time required to calculate connectivity *m*_*i*′*i*_ between several hundreds of patches prohibits the use of iterative algorithms to directly estimate the parameters of flexible (e.g. two-parameter) kernels. Thus, we developed an approach to approximate long-range (e.g. exponential-power) dispersal kernels. We defined *f*^2*D*^ as a mixture of *J* components:

$$\begin{array}{c} f^{2D}(\|x-y\|)=\sum_{j=1}^{J}[w_{j}f_{j}^{2D}(\|x-y\|)], \end{array}$$ (7)

where the *w*_*j*_ are positive mixture weights summing to 1, and 2*h*_*j*_ is the mean dispersal distance for exponential kernel $f_{j}^{2D}$ defined as:

$$\begin{array}{c} f_{j}^{2D}(\|x-y\|)=\frac{e^{-\|x-y\|/h_{j}}}{2\pi h_{j}^{2}}. \end{array}$$ (8)

Under this mixture formulation, the connectivity becomes:

$$\begin{array}{cc} m_{i^{\prime }i} & =\frac{\int_{x\in i^{\prime }}\int_{y\in i}\sum_{j=1}^{J}[w_{j}f_{j}^{2D}(\|x-y\|)]dydx}{A_{i^{\prime }}} \end{array}$$ (9)

$$\begin{array}{cc} & =\sum_{j=1}^{J}[w_{j}\frac{\int_{x\in i^{\prime }}\int_{y\in i}f_{j}^{2D}(\|x-y\|)dydx}{A_{i^{\prime }}}]. \end{array}$$ (10)

This formulation permits the connectivity of each mixture component *j* to be computed just once, since only the weights *w*_*j*_ require updating in an estimation procedure. We set $h_{j}=\frac{3}{2}\times 1.08^{j-1}$ (and *J* = 100), to obtain kernel components with mean distances ranging from 3 to 6110 m and higher resolution at smaller distances. To simplify parametrisation, and to avoid identifiability issues with the mixture of exponentials, we restrain weights using:

$$\begin{array}{c} w_{j}=P(\frac{j}{J}|s_{1},s_{2})-P(\frac{j-1}{J}|s_{1},s_{2}), \end{array}$$ (11)

where *P* is the cumulative distribution function of a beta distribution with parameters *s*_1_ and *s*_2_. We call any kernel of the form (Eq 7) using exponential kernels (Eq 8) weighted by (Eq 11) a beta-weighted mixture of exponentials (BWME) kernel.

In order to test whether BWME kernels provide a good approximation of other dispersal kernels, we fitted a BWME kernel to 3 standard [28] dispersal kernel types (exponential-power, power-law, and 2Dt), all with the same mean distance travelled (100 m). Model fitting was performed by minimizing the total absolute difference between the marginal cumulative distribution functions at 20,000 points spaced evenly between 0 and 1000 m. For each type of disperal kernel, 4 values of the shape parameter were tested.

### Bayesian estimation procedure

Among the four transition times, only *T*_*D*_ (i.e. the time when an infectious individual is detected) can be known precisely. Let ($t_{i,1},\cdots ,t_{i,k},\cdots ,t_{i,K_{i}}$) denote the set of *K*_*i*_ inspection dates in patch *i* (which may be partly censored by omissions in surveillance records). Let *p*(*T*_*D*,*i*_ = *t*_*i*,*k*_) denote the probability for an individual in patch *i* to be detected as infected at inspection date *t*_*i*,*k*_. Data provide the associated number $D_{i,k}^{+}$ of newly detected individuals, and the number $D_{i}^{-}$ of individuals upon which symptoms were not detected in any of the *K*_*i*_ inspections. These variables are modelled as:

$$\begin{array}{c} \left(D_{i,1}^{+},\cdots ,D_{i,K_{i}}^{+},D_{i}^{-}\right)∼\text{Multinomial}(N_{i},p\left(T_{D,i}=t_{i,1}\right),\cdots ,p\left(T_{D,i}=t_{i,K_{i}}\right),1-\sum_{k=1}^{K_{i}}p\left(T_{D,i}=t_{i,k}\right)), \end{array}$$ (12)

where *N*_*i*_ is the initial number of trees planted in patch *i*. A survival model [41] was used to derive *p*(*T*_*D*,*i*_ = *t*_*i*,*k*_) whilst accounting for censoring, imperfect detection sensitivity, and the expected dependencies between infections (Text A in S1 Texts). The probabilities *p*(*T*_*D*,*i*_ = *t*_*i*,*k*_) were determined from the set of model parameters Θ, using a smoothed representation of the expected epidemic, and were not conditioned on past observations. Thus, Eq (12) provides a pseudo-likelihood for the observed data (Text A in S1 Texts). Based on this pseudo-likelihood, Bayesian inference (for parameter set Θ) was performed via Markov chain Monte Carlo (MCMC) using a Gibbs sampler with embedded adaptive Metropolis-Hastings steps and data augmentation for the unknown planting and inspection dates (Texts B and C in S1 Texts). By data augmentation, we mean the explicit introduction of latent variables [42–44].

### Estimation for simulated epidemics

To assess the accuracy (i.e. amount of bias) and precision (i.e. amount of variance) of the estimation of dispersal parameters, 10 epidemics were simulated under each combination of 7 disease introduction scenarios × 3 dispersal kernels × 4 parameter estimation scenarios. All simulations were performed under the same virtual landscape derived from the surveillance database: we retained the spatial coordinates (and thus the geometry) of the patch polygons, but all other potential spatio-temporal dependencies were suppressed through the random permutation of orchard-level data including planting densities and patch planting/removal/replanting dates. When density or planting date were missing in the database, their values were drawn from the corresponding empirical distribution. Simulations were performed with 1 time step per day, and 1 survey per patch per year, with inspection days drawn from the corresponding empirical distribution. The transmission coefficient *β* was fixed at 1.5 (which leads to realistic epidemic dynamics) and all other parameters were fixed at the expected values of their prior distributions (Text B in S1 Texts).

The three simulated kernels correspond to short-, medium- and long-range dispersal. They were parametrised using low-dimension mixtures of exponential kernels (Eq 7) with fixed mean distances and weights (Table 1, mixture parameters). These were subsequently approximated by the BWME kernel minimizing the Kullback-Leibler (KL) distance [45] between the two probability density functions (Table 1, simulation parameters).

**Table 1:** Parameters of the three dispersal kernels used in the simulation study.

Kernel range	Simulation parameters		Mixture parameters	
	*s*_1_	*s*_2_	*J*	Mean distances in m (weights)
short	12727.3	29264.2	1	25 (1)
medium	9.3	18.1	2	25 (2/3), 100 (1/3)
long	5.5	8.4	3	25 (3/6), 100 (2/6), 300 (1/6)

Epidemics were simulated using BWME kernels with parameters *s*_1_ and *s*_2_ (left), approximating exponential mixture kernels with *J* mixture components (right).

The seven introduction scenarios were defined by the following number of introduction patches (and the initial prevalence *p*_*i*_ in these patches): 1 (25%), 5 (10%), 10 (5%), 15 (2%), 20 (1%), 25 (1%) or 30 (1%). For a given introduction scenario, all simulations were performed with the same introduction patches, which were chosen at random with the constraint that the first introduction occurred at year 1 and all other introductions occurred before year 6 (S1 Fig).

In order to identify whether our MCMC estimation procedure (Text C in S1 Texts) encountered identifiability issues with some parameters, we tested 4 estimation scenarios targeting parameter sets of increasing size (Table 2), with all other parameters fixed at the values used for simulation.

**Table 2:** Parameter sets for four estimation scenarios.

Parameter	Definition	Θ_1_	Θ_2_	Θ_3_	Θ_4_
*β*	transmission coefficient	✔	✔	✔	✔
*μ*	= *s*_1_/(*s*_1_+*s*_2_); mean of kernel weight distribution	✔	✔	✔	✔
*σ*	= *s*_1_+*s*_2_; shape of kernel weight distribution	✔	✔	✔	✔
*ρ*	detection sensitivity	-	✔	-	✔
*θ*_1_	shape of latent period distribution	-	-	✔	✔
*θ*_2_	scale of latent period distribution	-	-	✔	✔

For each estimation scenario, the set of parameters to be estimated, Θ, comprises the parameters indicated with a ✔.

Both simulated epidemics and the smoothed epidemics of the pseudo-likelihood started at the beginning of year 1 and stopped at the end of year 22. Because some MCMC chains became trapped in local maxima associated with negligible likelihoods, we performed 10 MCMC chains under each estimation scenario (applied to each simulated epidemic), which produced 8400 MCMC chains in total. Within each combination of epidemic replicate × kernel × introduction × estimation scenario, we retained the MCMC chain with the highest mean posterior log-likelihood. Then, for each of these 840 chains, indices of accuracy (resp. precision) were defined as the mean (resp. span of the 95% credibility interval) of the posterior KL distances between the probability density functions *f*^2*D*^ (Eq 7) of simulated and estimated kernels. For ease of interpretation, simulated and estimated kernels were plotted using the distribution function of the distance travelled:

$$\begin{array}{c} F^{1D}(\|x-y\|)=\sum_{j=1}^{J}(w_{j}[1-(1+\frac{\left\|x-y\right\|}{h_{j}})e^{-\frac{\left\|x-y\right\|}{h_{j}}}]). \end{array}$$ (13)

This function is the cumulative version of the 1-dimensional *f*^1*D*^ (i.e. the probability density function of the distance travelled), which is obtained by integrating (marginalising) *f*^2*D*^ (Eq 7) over all directions.

Finally, to assess the impact of detection sensitivity (*ρ*) on the accuracy and precision of the estimation of the dispersal kernel, we performed an additional simulation-estimation study. For 99 equally spaced values of *ρ* between 0.01 and 0.99, a unique epidemic was simulated. Each epidemic started at year 1 from a single introduction patch with 25% prevalence, and spread under the long-range kernel scenario (Table 1). Default values were used for all other parameters. For each of the 99 simulated epidemics, independent estimations were carried out under the most exhaustive scheme (Θ_4_) with 3 prior distributions for detection sensitivity *ρ* corresponding to different levels of available prior information (Text B in S1 Texts). For each combination of prior × detection sensitivity, 10 MCMC chains were run, leading to 2970 MCMC chains. For each value of *ρ*, posterior distributions were inferred using all chains with non-negligible mean posterior likelihood.

### Estimation for a real epidemic

Using PPV surveillance data, estimation was carried out under the most exhaustive scheme (Θ_4_) to infer parameters of the spatial *SEHDR* model. As above, and for the same reasons, we ran multiple MCMC chains and retained the chain with the highest mean posterior log-likelihood (Text C in S1 Texts). The number of introduction patches *κ* was fixed at integer values in the range 1-24, and 30 chains were run per fixed *κ*. This approach was taken because each unit increase in *κ* adds two parameters (additional introduction patch identity and initial prevalence) to Θ, which always increases the posterior log-likelihood (various uninformative and weakly informative priors were tested). Thus, to avoid over-fitting, identification of *κ* was treated as a model selection problem for which we maximised the Fisher information criterion I(κ) (Text D in S1 Texts).

## Results

### Impact of parameter values on simulated epidemics

**Fig 2:**
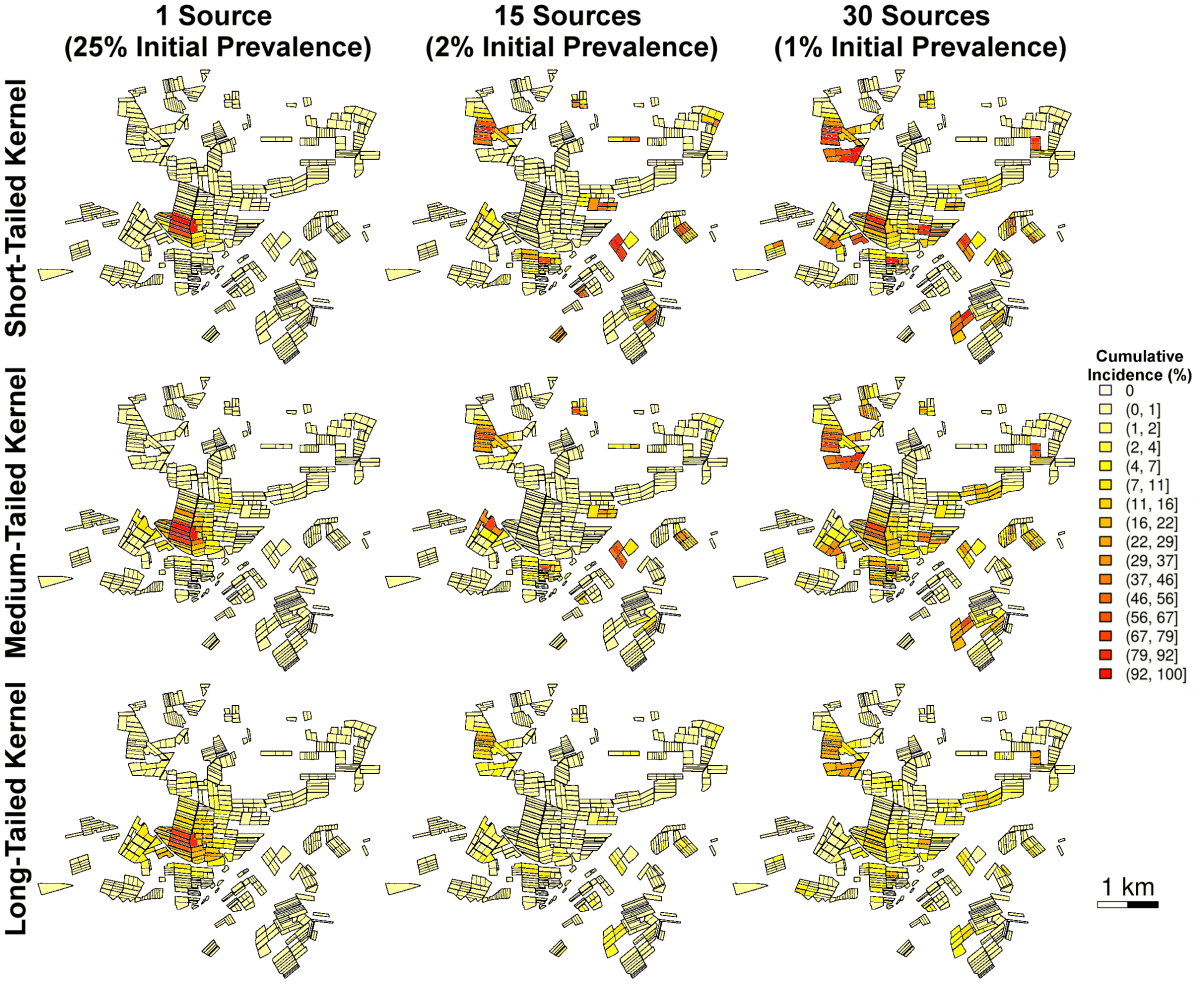
Cumulative detected incidence at the end of year 22 for nine simulated epidemics. Each polygon represents one peach orchard. From left to right, the number of introduction patches (with initial disease prevalence) are: 1 (25%), 15 (2%) and 30 (1%). From top to bottom: simulations generated under short-, medium- and long-range kernel scenarios.

The parameter combinations chosen to test the inference procedure cover a wide range of epidemic behaviour, from local to widespread epidemics and from low to high incidence (Fig 2). The general trends are that the stochastic variability has less effect than the introduction scenario or kernel type, that more introduction patches generally lead to more widespread epidemics, and that higher disease prevalence in the introduction patches does not necessarily increase the final local cumulative incidence (S2 and S3 Figs). Increasing kernel range generally decreases the cumulative incidence (S2 and S3 Figs), especially near the introduction patches, although these epidemics are more widespread (Fig 2).

### Evaluation of the estimation procedure

A key inovation in our estimation procedure is the BWME dispersal kernel. This kernel provides close approximations to exponential-power and power-law kernels for all tested values of the shape parameter (S4 and S5 Figs). Such flexibility is an interesting property when one does not know which kernel type to assume, which is a common issue. However, the fit to the 2Dt kernels was more approximate (S6 Fig). This is not surprising since the 2Dt kernel is essentially a continuous mixture of Gaussians. Thus, switching the basis functions from exponential to Gaussian (giving a BWMG kernel) may greatly improve the fit.

**Fig 3:**
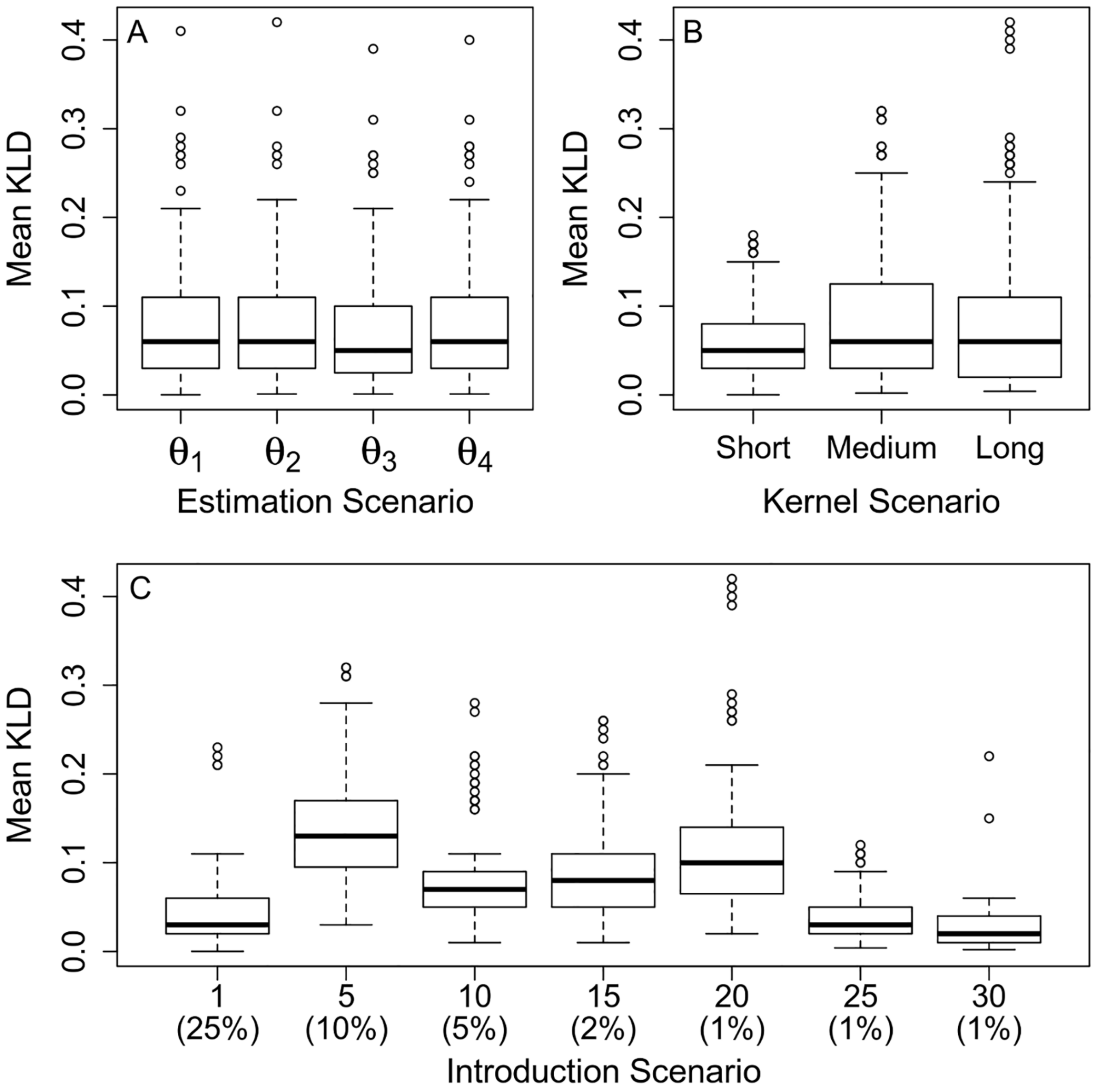
Boxplots of distances between simulated and estimated dispersal kernels. Impact of (A) estimation scenario, (B) kernel range, and (C) disease introduction scenario [number of introduction patches (with initial disease prevalence)] on the accuracy of estimated dispersal kernels. Accuracy is measured by the Kullback-Leibler distance (KLD) between simulated and estimated dispersal kernels. Each panel consists of 840 points, which correspond to 10 epidemics × 7 disease introduction scenarios × 3 dispersal kernels × 4 parameter estimation schemes.

The distribution of Kullback-Leibler (KL) distances between simulated and estimated kernels demonstrates that estimation accuracy is not affected by the inclusion of sensitivity and latent period parameters in the estimation scheme (Fig 3A). Neither is the median accuracy of the estimated kernels affected much by the range of the dispersal kernel (Fig 3B). However, for longer-range dispersal kernels, KL distances can become more extreme (Fig 3B), and the span and variance of their 95% credibility intervals increase (S7B Fig). This shows that the precision of the estimated kernel decreases with increasing dispersal range. The most influential factor on the accuracy and precision of estimated dispersal kernels is the introduction scenario (Fig 3C and S7C Fig). However, the effect of the introduction scenario is neither strongly related to the number of introduction patches nor to the associated initial prevalence, but rather to the presence of an introduction patch in the dense central cluster of patches (Fig 3 and S1 Fig). The impact of kernel range and introduction scenario on kernel estimation can also be seen by the visual comparison between simulated and estimated kernels (S8, S9 and S10 Figs).

**Fig 4:**
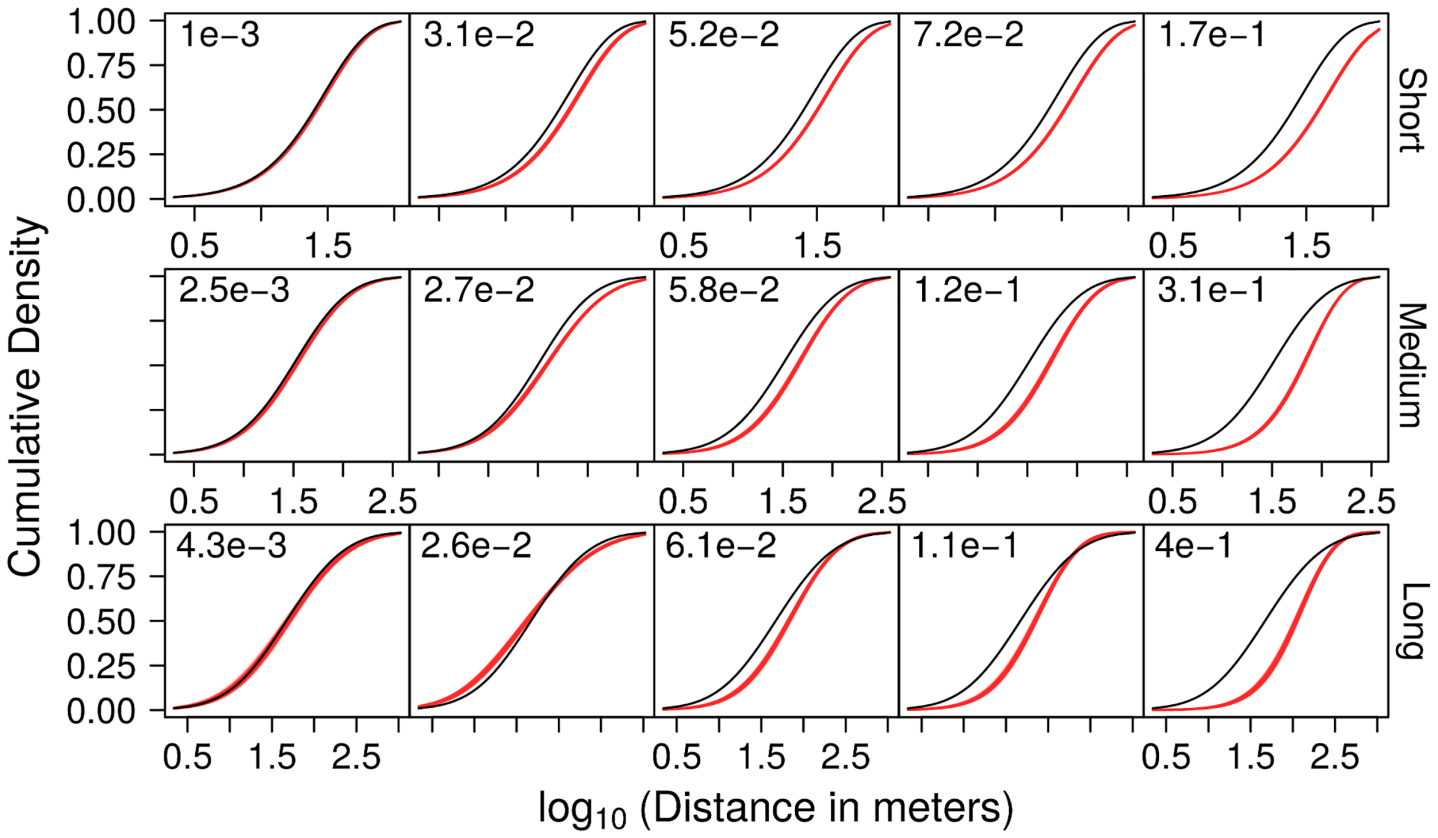
Comparison of simulated and estimated dispersal kernels. From left to right: kernels with the minimum, lower quartile, median, upper quartile and maximum Kullback-Leibler (KL) distances (posterior mean), as estimated (red) under the most exhaustive scheme (Θ_4_), based on simulated epidemics with short-, medium- and long-range kernels (from top to bottom; black). Kernels are represented by their marginal cumulative distribution function *F*^1*D*^ (with distance from the source represented on the log_10_ scale). The mean KL distance is indicated for each estimation.

For each of the 3 simulated kernels, the distribution of KL distances was summarised by its minimum, quartile and maximum values across all 7 introduction scenarios × 10 epidemics per scenario. The comparison between simulated kernels and their estimates within the most exhaustive scheme (Θ_4_) shows that the 3 kernels are very accurately estimated for some simulated epidemics (left column in Fig 4 and S11 Fig). However, dispersal distances are often overestimated, with the median KL distance increasing from 5.2×10^−2^ to 6.1×10^−2^ with increasing kernel range. A closer look at the estimation curves corresponding to the median KL distance reveals that estimated distances do not exceed the simulated distances by more than 0.25 on the log_10_ scale. Dispersal distances are thus overestimated by a factor below 1.8 (1.2 for the mode; see central column in S11 Fig). Even for the most challenging of the 70 epidemics simulated with the long-range dispersal kernel (bottom-right panel in Fig 4 and S11 Fig), the difference between the two curves remains below 0.6 on the log_10_ scale. This value translates into less than 4-fold estimation errors (less than 4.3 for the mode; see right column in S11 Fig), which is high but still within one order of magnitude. By contrast, precision is very high for all kernel ranges, as indicated by a median span below 0.04 for the 95% posterior credibility interval of KL distances (S7 Fig) and the corresponding overlapping red lines in each plot of Fig 4 and S11 Fig.

The estimated values of the other parameters are generally close to the values used for simulation, but the relative bias varies among parameters, kernel ranges, and introduction scenarios (S12 Fig). Detection sensitivity (*ρ*) is the most precisely estimated parameter, followed by the shape of the latent period (*θ*_1_) for which the estimates are also almost unbiased. Bias can be more severe for the scale of the latent period (*θ*_2_) and the transmission coefficient (*β*), with up to 45% under- and over-estimation (respectively) in the worst-case combinations of kernel and introduction scenarios (S12 Fig, top row for *θ*_2_ and bottom row for *β*). For these two parameters, the impact of the introduction scenario on parameter estimation increases with kernel range.

The simulation-estimation study on *ρ* shows that the estimation procedure is robust to detection sensitivities below the default value (0.8) used in the rest of this work. Indeed, although reducing *ρ* reduces (by definition) the proportion of detected cases, the link between detection and epidemic control results in a disproportionate increase in the total number of infected hosts as *ρ* decreases, providing more data for statistical inference–except when *ρ* reaches extremely small values (S13 Fig). As a result (see S14 and S15 Figs): (i) accuracy of kernel estimation is not reduced as detection sensitivity decreases; (ii) precision of kernel estimation is only affected when *ρ* is very close to 0 or 1; (iii) increasing the precision of the prior on *ρ* only affects the accuracy of kernel estimation for *ρ*>0.8 (i.e. when epidemic size–and thus data available for inference–is strongly reduced by effective control). Finally, we note that stochastic variations among replicated epidemics have more influence than *ρ* on the KL distance between simulated and estimated kernels (S15 Fig).

### Estimation for a real epidemic

**Fig 5:**
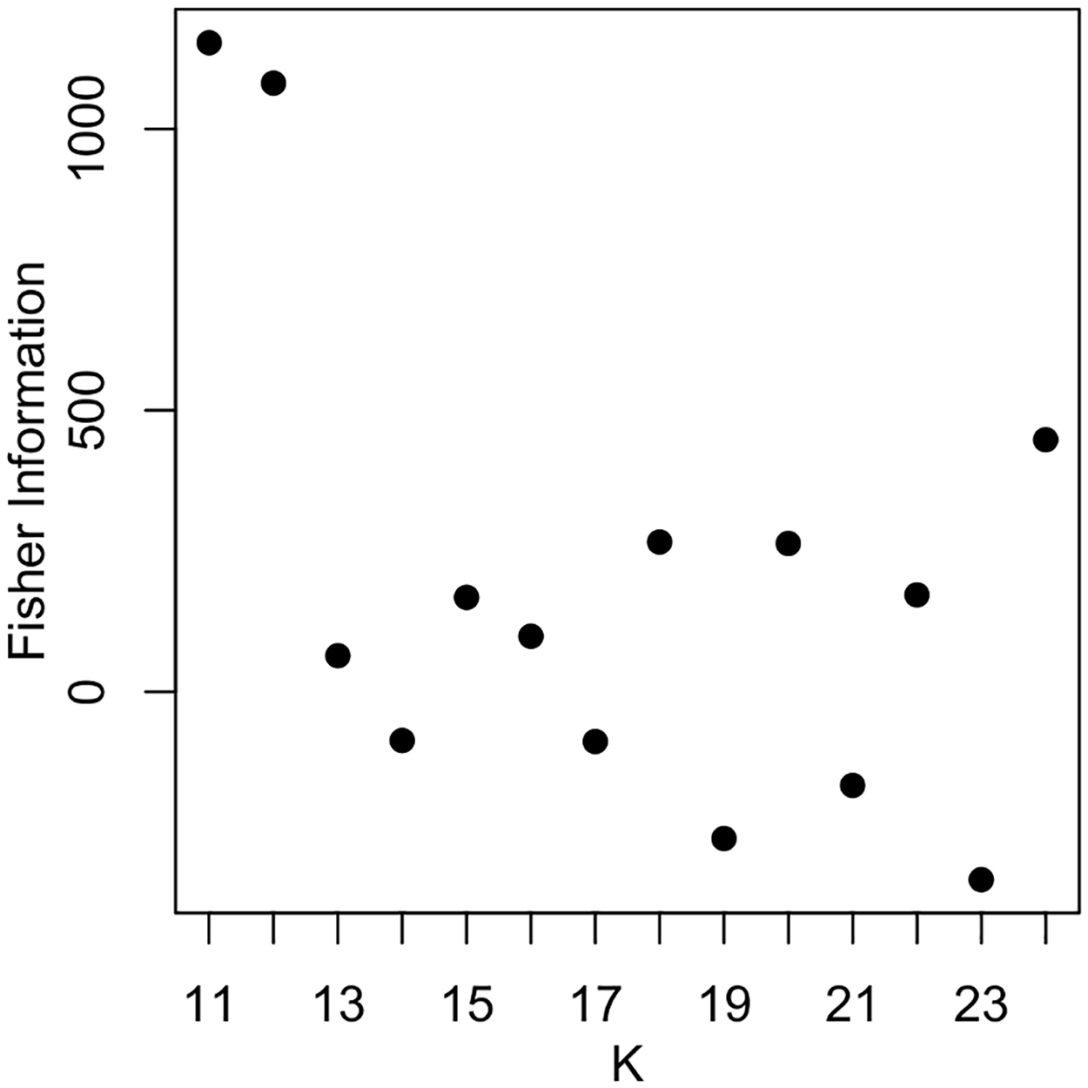
Impact of the number of introduction patches (*κ*) on the expected Fisher information for the sharka epidemic. For each *κ*, the estimation with the highest mean posterior log-likelihood was retained. For *κ*<10 no introduction patch combination returned a finite posterior log-likelihood. The empirical approximation of the Fisher information was maximal at *κ* = 11.

Once validated on simulated epidemics, we used the developed inference framework to estimate the dispersal kernel of *Plum pox virus* (and thus of the flight distances of the infectious aphid vectors) based on survey data. As a first step, we inferred the number of introduction patches. For *κ*<10, no combination of introduction patches returned a finite posterior log-likelihood. The Fisher information criterion was maximised at *κ* = 11 (Fig 5), indicating that improvement in model fit saturates beyond this point. This suggests that the most robust inference is obtained with *κ* = 11. These 11 introduction events among 547-579 orchards planted over 22 years (planting date is unknown for 32 orchards) correspond to disease introduction probabilities of 0.5 per year and 1.90-2.01×10^−2^ per orchard planted.

**Fig 6:**
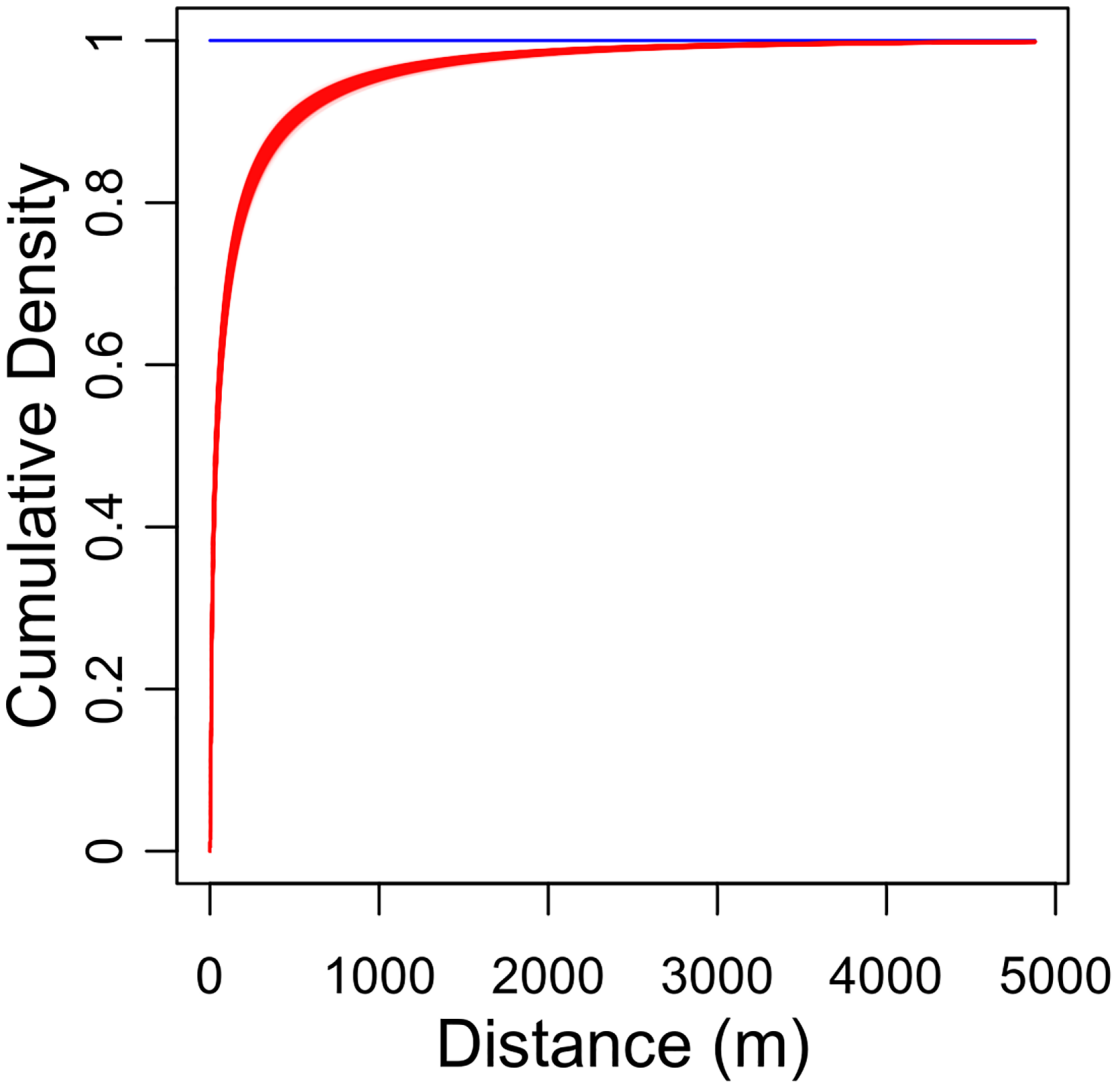
Estimated dispersal kernel for the sharka epidemic. The posterior marginal cumulative distribution function, *F*^1*D*^, of the fitted dispersal kernel, obtained for *κ* = 11 (i.e. the number of introduction patches maximising the Fisher information). The plotted posterior distribution was obtained from 4000 MCMC samples. One line is plotted per sample.

Summary statistics of the posterior distributions of key parameters and percentiles of the dispersal kernel were tabulated for *κ* = 11 (Table 3). From the estimated values of *s*_1_ and *s*_2_, we derived the weights of the kernel components (S16 Fig), the dispersal kernel, the cumulative distribution function (Fig 6) and the probability density function (S17 Fig) of aphid flight distances. These figures, and the estimated quantiles shown in the second part of Table 3, demonstrate the substantial contribution of long-range dispersal to aphid-borne virus epidemics. Indeed, almost 50% of the infectious aphids leaving a tree land beyond 100 m (median distance = 92.8 m; CI_95%_ = [82.6-104 m]), and nearly 10% land beyond 1 km (last decile = 998 m; CI_95%_ = [913-1084 m]).

**Table 3:** Summary statistics for parameters estimated from the survey data.

	Mean	SD	TSSEM	CI_95%_
*β*	1.32	2.80 × 10^−2^	7.1 × 10^−4^	1.27-1.38
*s*_1_	2.32	1.11 × 10^−1^	2.4 × 10^−3^	2.11-2.55
*s*_2_	2.45	8.66 × 10^−2^	1.5 × 10^−3^	2.29-2.62
*ρ*	0.659	7.73 × 10^−3^	1.9 × 10^−4^	0.643-0.674
*θ*_*exp*_	1.92	8.74 × 10^−2^	2.2 × 10^−3^	1.75-2.09
*θ*_*var*_	0.442	8.69 × 10^−2^	2.0 × 10^−3^	0.291-0.631
*d*_5%_	5.0	3.23 × 10^−1^	7.2 × 10^−3^	4.4-5.7
*d*_10%_	8.9	5.98 × 10^−1^	1.3 × 10^−2^	7.8-10.1
*d*_50%_	92.8	5.47	1.2 × 10^−1^	82.6-104
*d*_90%_	998	4.35 × 10^1^	7.7 × 10^−1^	913-1084
*d*_95%_	1742	7.20 × 10^1^	1.2	1604-1887

Summary statistics including the posterior mean, standard deviation (SD), time-series standard error of the mean (TSSEM) and 95% credibility intervals (CI_95%_) are reported for the transmission coefficient *β*, the kernel parameters *s*_1_ and *s*_2_, the detection sensitivity *ρ*, the expected duration *θ*_*exp*_ = *θ*_1_×*θ*_2_ of the latent period and associated variance $\theta_{var}=\theta_{1}\times \theta_{2}^{2}$. Posterior distributions of the 5^th^, 10^th^, 50^th^, 90^th^ and 95^th^ percentiles of aphid flight distances *d* are also summarised.

## Discussion

In this work, we developed a spatially-explicit Bayesian inference framework for the estimation of disease dispersal parameters when surveillance data are gathered at the patch level. The simulation and inference procedures take into account that disease status assessment is incomplete because surveillance has an imperfect detection sensitivity and a finite spatio-temporal coverage. We assessed the quality of the inference procedure through comparison between parameter values used for simulation and corresponding estimates. Then, we applied this approach to *Plum pox virus* surveillance data, to obtain the first estimate of an aphid dispersal kernel at the landscape scale. We discuss below the interest and limitations of the proposed approach and results.

### Sources of uncertainty and model validation

Since the dispersal kernel is the key component of spatial epidemiological models, we focused attention on its estimation and treated the other parameters as nuisance parameters (i.e. parameters than are inferred to limit bias in the estimation of the distribution of interest). S12 Fig shows how simulated and estimated values compare for all nuisance parameters. Recent methodological advances have permitted the extraction of crucial information on the dispersal kernel of four plant diseases from surveillance data [13, 16–18] and observational studies [15, 30]. These estimation procedures all account for unobserved infection times, with additional methodological challenges related to large heterogeneous landscapes [16], introduction from external sources [17, 18], or active disease control [18]. The present work handles these various processes and, contrary to the abovementioned studies which all assume a known detection sensitivity, also accounts for this poorly known variable which adds a layer of uncertainty into the surveillance process. Inclusion of parameters for detection sensitivity and the latent period in the estimation procedure (Table 2) barely affects the KL distance between simulated and estimated kernels (Fig 3 and S7 Fig); hence the inclusion of these extra parameters during inference based on PPV surveillance data. The resulting estimate of detection sensitivity is *ρ* = 0.66 (Table 3). Although a previous analysis showed that the presence of undetected infectious individuals resulted in slightly overestimated dispersal distances [19], here we show that our estimation procedure is robust to detection sensitivities far below one, even with weak prior information on *ρ* (S14 and S15 Figs). The dataset used for inference contains information on the disease status of more than 401,000 trees over 15 years, and is associated with a substantial level of censoring (on the dates of planting, inspection, infection, end of the latent period, and removal). For these reasons, using data augmentation to infer the transition times was unlikely to scale successfully to our analysis. Instead, we used a pseudo-likelihood where the unknown numbers of infectious and removed trees were replaced by their expected values. Intuitively, this approach can be expected to work best in highly connected landscapes, where epidemics are more likely to follow their expected course, and to become more erroneous in patchy landscapes where stochastic events can deflect epidemics away from their expected course. This might explain in part why the smaller KL distances in Fig 3C correspond to those introduction scenarios where a source patch was located in the most highly connected region of the study area.

A unique feature of the present work is the validation of the estimation of the dispersal kernel through comparing known functions used in simulations and the corresponding functions estimated from these simulated epidemiological data sets. Although this is an intuitive and standard practice [46–48], previous estimations of plant disease dispersal parameters instead used goodness-of-fit statistics between actual and simulated spatiotemporal patterns as a way to validate their inference models [16–18]. This general trend to rely on goodness-of-fit statistics, without performing simulation-based validation tests, may be due to the high computational burden associated with such validation procedures which require several simulation scenarios and several independent estimations per scenario to assess the accuracy and precision of the estimation algorithms. Since we focus on dispersal kernel estimation, rather than on model predictions as in [16, 17], simulation-based validation was useful to demonstrate that, despite the approximations of the pseudo-likelihood, dispersal kernel estimation was generally very precise. Accuracy was often high for short-range kernels, and dispersal distance estimates ranged from very accurate to overestimated for longer-range kernels (Figs 3 and 4). The same approach also showed that both the precision and the accuracy of dispersal kernel estimation is unaltered when the probability *ρ* to detect a symptomatic/infectious tree is in the range 0.05–0.8 (S15 Fig).

The observed overestimation is not likely to be caused by insufficient flexibility in the BWME kernel because, even for the 2Dt dispersal kernel (which the BWME kernel does not fit perfectly), the magnitude of the difference between the two kernels is negligible in comparison with the difference between simulated and estimated kernels. It is not likely either to be caused by choosing the MCMC chain with the highest mean posterior likelihood (among 10 chains) since this procedure was just used to remove degenerate chains (and coherence between all other chains was high). Although this procedure is rather wasteful of problem-free chains, and provides lower precision than alternative approaches to multi-chain analysis, there is no reason to expect any bias concerning the mean (or other statistics) of the posterior distribution. It is most likely that the estimation bias reported here arose from approximations made (for practical reasons) within the pseudo-likelihood.

### Dispersal in a patchy landscape

Our inference procedure explicitly accounts for patch geometry and patch-level aggregation of surveillance data. Although this choice was data-driven (infected tree numbers–not individual locations–were included in the database), for landscape-scale studies this approach appears to strike an interesting compromise between computational feasibility and spatial realism. Indeed, considering the disease status of over 401,000 individuals simultaneously would cause major computational issues given the size of the resulting connectivity matrix. Conversely, spatial models commonly use the coordinates of patch centroids in connectivity calculations (e.g. [14, 19]). However, this neglects patch geometry and can be expected to bias connectivity estimates (i) when patch shapes and sizes are disparate, or (ii) when patch dimensions are of the same order of magnitude as the distances between patches. To exemplify (i), consider a small patch located next to a large patch, where many of the propagules leaving the small patch can be expected to land, but a much lower proportion of the propagules leaving the large patch are expected to fall in the small patch. To exemplify the importance of (ii), consider that many more propagules can be exchanged between two large adjacent orchards than would be calculated using the distance between their distant centroids. Although our approach neglects the effects of disease aggregation within patches, it does account for patch size and geometry that both impact disease spread [49]. The use of Eq (6) to integrate patch geometry, combined with the BWME kernel, can thus be useful for the inference of the landscape-scale dispersal kernels of many wind- and vector-borne diseases.

A rigourous assessment of connectivity between patches is also necessary because of its influence on parameter estimation. Our study shows that kernel range affects both the KL distance between simulated and estimated dispersal kernels (Fig 3 and S7 Fig) and the cumulative incidence (S2 and S3 Figs). This pattern reflects how parameter identifiability depends on statistical power, which depends on cumulative disease incidence, which in turn depends on landscape connectivity. Short-range kernels imply greater local connectivity than long-range kernels, leading to relatively intense local transmission but reduced transmission at greater distances. Whether or not shorter-range kernels generate larger epidemics depends on the proportion of potential transmission events falling outside host patches, and thus on landscape configuration. Here, larger cumulative incidences were obtained using smaller kernels because, in our patchy agricultural landscape, many dispersal events generated by long-tailed kernels do not end within host patches.

### Impact of disease introductions on inference

Disease introduction scenarios had a substantial effect on the accuracy and precision of the inferred dispersal kernel (Fig 3 and S7 Fig). Surprisingly, this effect does not seem related to either the number of introduction patches or the associated initial prevalence. However, we note that lower KL distances between simulated and estimated dispersal kernels (in introduction scenarios 1, 6 and 7) are associated with introductions occurring in the highly connected central patches (S1 Fig). The resulting higher cumulative incidence probably improves estimation for the reasons given above.

During parameter estimation, we did encounter multi-modality in the posterior likelihood surface, which may arise when fitting ecological dynamic models to data, even without observation error and model mis-specification [50]. For epidemic scenarios with both a short-range kernel and a high number of introduction events, misidentifying some of the introduction patches had a large negative effect on the likelihood, and some MCMC chains were trapped in degenerate solutions. For this reason, we ran the MCMC algorithms many times and carefully compared the posterior likelihoods and parameter estimates of all chains before making inference. We also considered alternative algorithms such as parallel tempering [51] or equi-energy sampling [52], which increase the likelihood of between-mode transitions. However, the extra computational burden of these approaches was considered superfluous given that the observed differences in the posterior likelihoods of various modes were typically relatively large. Thus, launching a large number of chains to increase the likelihood of identifying the global mode was a reasonable compromise. We have extensively tested this approach, reporting here the results of several thousand MCMC chains, and have found that in practice results are consistent.

Overall, inference of epidemiological parameters is easier for epidemics where disease introductions are well characterized, or at least infrequent. Unfortunately, this was not the case with the PPV-M dataset, and estimating the number of introduction patches *κ* was challenging. Such difficulty is by no means unique to the current study (see e.g. [17]). Reversible-jump MCMC (RJMCMC) [53] is a method for performing MCMC when the dimension of the parameter space is unknown and inferred from data. We initially attempted various implementations of RJMCMC, but found it impossible to construct priors that could both prevent over-fitting and provide robust posterior probabilities for *κ* under a wide variety of epidemiological scenarios. To circumvent this issue we inferred *κ* based on the Fisher information. This gives a minimum-variance estimator that provides robust inference with a good balance between under- and over-fitting–although it does not permit the estimation of posterior probabilities associated with the various *κ*. This approach has been used successfully in similar situations [54].

### Insights into aphid biology

Like most plant viruses, PPV is transmitted by winged non-colonising aphids in a non-persistent manner [33]. To match the characteristics of this widespread transmission process, in our model transmission events are independent (conditional on infection sources) and transmission distances directly depend on host locations and on the distance travelled by an aphid within a single infectious flight. Although estimating this aphid dispersal kernel is crucial to plant virus epidemiology, it has long remained elusive. Traditional ecological methods such as capture-mark-recapture provide little information regarding aphid dispersal at the landscape scale [32]. This has been a major obstacle to the parametrisation of models simulating the dispersal of these vectors and the pathogens they spread, as exemplified by the scarcity of landscape-scale models on cereal aphids [55] and by the informed guesses of flight-distance parameters in such models [56]. Here we estimated, for the first time, the dispersal of aphid vectors at the landscape scale. This estimation indicates that 50% of the infectious aphids leaving a tree land within about 90 meters, while about 10% of flights terminate beyond 1 km. Although dispersal estimation from simulated epidemics suggests that these distances may be overestimated, the large number of flights estimated to terminate within some tens of meters of the source tree is consistent with previous studies of within-patch clustering of trees infected by PPV-M [33, 57, 58] or PPV-D [38, 59]. Indeed, one of these studies [38] shows that 50% of the new PPV cases occur within 35-70 m of the nearest previous case; in addition, 10% of the new PPV cases were found beyond 200-460 m from the nearest previous case. Although the proportion of new PPV cases captured within a given radius is not equivalent to a dispersal kernel (e.g. because the trees are not always infected by the nearest previously detected neighbour), the figures are of the same order of magnitude. In particular, both studies highlight the long range of the dispersal kernel. Our estimation of the dispersal kernel at the landscape scale has important consequences. For example, current French regulations enforce at least one visual inspection per year within 2.5 km of a detected sharka case (followed by the removal of all trees with sharka symptoms). Our results suggest that less than 3% of flights should thus go beyond this radius (Fig 6). In a patchy French landscape, most of these aphids would land outside a peach orchard and thus lead to no infection. Such procedures are thus likely to efficiently detect most of the aphid-mediated secondary infections; actually, given the cost of surveillance and the speed of disease spread, this radius may even be oversized. Future work based on this study could aim at the definition of new management strategies against PPV. More generally, our results provide a unique reference point on the epidemiology, simulation and control of the principal group of plant viruses (i.e. those caused by non-persistant aphid-borne viruses), which have a major epidemiological and economic impact. Finally, by focusing on incidence data the presented estimation approach is adaptable to many epidemiological situations, including other vector-borne and airborne fungal diseases.

## Supporting information

S1 Fig Simulated planting years and introduction patch locations used in the simulation study. The first map (top left) represents the randomisation of the first planting years of the 553 patches. These years were sampled without replacement from their empirical distribution. The other maps show the location and planting year of each introduction patch in the seven introduction scenarios. The number of introduction patches and their initial prevalence are indicated for each introduction scenario. Note the greater landscape connectivity in the central area.
(TIFF)

S2 Fig Cumulative detected incidence for the introduction scenarios tested in the simulation study. For each introduction scenario, the number of introduction patches and the corresponding initial disease prevalence are mentionned above the graph. The three tested kernels are represented by different colours. For each combination of kernel and introduction scenarios, 10 independant simulated epidemics are shown.
(TIFF)

S3 Fig Cumulative detected incidence for the kernels tested in the simulation study. For each kernel, the seven tested introduction scenarios are represented by different colours. For each combination of kernel and introduction scenarios, 10 independant simulated epidemics are shown.
(TIFF)

S4 Fig Best-fit BWME kernel approximations of exponential-power kernels. The kernels corresponding to 4 values of the shape parameter are represented by their cumulative distribution function *F*^1*D*^ (top) and the associated probability density function *f*^1*D*^ (bottom) of the distance travelled. Green dashed line: mean distance travelled.
(TIFF)

S5 Fig Best-fit BWME kernel approximations of power-law kernels. The kernels corresponding to 4 values of the shape parameter are represented by their cumulative distribution function *F*^1*D*^ (top) and the associated probability density function *f*^1*D*^ (bottom) of the distance travelled. Green dashed line: mean distance travelled.
(TIFF)

S6 Fig Best-fit BWME kernel approximations of 2Dt kernels. The kernels corresponding to 4 values of the shape parameter are represented by their cumulative distribution function *F*^1*D*^ (top) and the associated probability density function *f*^1*D*^ (bottom) of the distance travelled. Green dashed line: mean distance travelled.
(TIFF)

S7 Fig Boxplots of the variation among estimated dispersal kernels. Impact of (A) estimation scenario, (B) kernel range, and (C) disease introduction scenario [number of introduction patches (with initial disease prevalence)] on the precision of estimated dispersal kernels. Precision is measured by the span of the 95% credibility interval of Kullback-Leibler distances (Span KLD) between simulated and estimated dispersal kernels. Each panel consists of 840 points, which correspond to 10 epidemics × 7 disease introduction scenarios × 3 dispersal kernels × 4 parameter estimation schemes.
(TIFF)

S8 Fig Influence of introduction scenarios on the estimation of a short-range dispersal kernel. For each introduction scenario, 10 epidemics were simulated with a short-range kernel (black dashed curve), and 10 MCMC chains were run per simulated epidemic. The posterior distributions of the kernel obtained under the most exhaustive estimation scheme (Θ_4_) are represented for all chains with non-negligible mean posterior likelihood. The proportion of MCMC chains with negligible mean posterior likelihood (mean proportion: 10%) increases quadratically with the number of source orchards. Kernels are represented by their marginal probability density function *f*^1*D*^ (top row), and by their marginal cumulative distribution function *F*^1*D*^ with the distance from the source represented on the natural scale (middle row) or on the log_10_ scale (bottom row).
(TIFF)

S9 Fig Influence of introduction scenarios on the estimation of a medium-range dispersal kernel. For each introduction scenario, 10 epidemics were simulated with a medium-range kernel (black dashed curve), and 10 MCMC chains were run per simulated epidemic. The posterior distributions of the kernel obtained under the most exhaustive estimation scheme (Θ_4_) are represented for all chains with non-negligible mean posterior likelihood. The proportion of MCMC chains with negligible mean posterior likelihood varies among introduction scenarios, with a mean proportion of 2.6%. Kernels are represented by their marginal probability density function *f*^1*D*^ (top row), and by their marginal cumulative distribution function *F*^1*D*^ with the distance from the source represented on the natural scale (middle row) or on the log_10_ scale (bottom row).
(TIFF)

S10 Fig Influence of introduction scenarios on the estimation of a long-range dispersal kernel. For each introduction scenario, 10 epidemics were simulated with a long-range kernel (black dashed curve), and 10 MCMC chains were run per simulated epidemic. The posterior distributions of the kernel obtained under the most exhaustive estimation scheme (Θ_4_) are represented for all chains with non-negligible mean posterior likelihood. The proportion of MCMC chains with negligible mean posterior likelihood is low (mean proportion: 0.4%) for all the introduction scenarios. Kernels are represented by their marginal probability density function *f*^1*D*^ (top row), and by their marginal cumulative distribution function *F*^1*D*^ with the distance from the source represented on the natural scale (middle row) or on the log_10_ scale (bottom row).
(TIFF)

S11 Fig Comparison of simulated and estimated dispersal kernels. From left to right: kernels with the minimum, lower quartile, median, upper quartile and maximum Kullback-Leibler (KL) distances (posterior mean), for all chains with non-negligible mean posterior likelihood. Estimations (red) under the most exhaustive scheme (Θ_4_) are based on simulated epidemics with short-, medium- and long-range kernels (from top to bottom; black). Kernels are represented by their marginal probability density function *f*^1*D*^. The mean KL distance is indicated for each estimation.
(TIFF)

S12 Fig Comparison of simulated and estimated nuisance parameters. For each combination of short-, medium- and long-range kernels (from top to bottom) and introduction scenarios (colour-coded as in S3, S8, S9 and S10 Figs), 10 epidemics were simulated and 10 MCMC chains were run per simulated epidemic. The curves represent the posterior distribution of the parameters obtained under the most exhaustive estimation scheme (Θ_4_) for all chains with non-negligible mean posterior likelihood. Dashed lines: parameter values used in the simulations.
(TIFF)

S13 Fig Cumulative detected incidence at the end of year 22 across the range of detection sensitivities (*ρ*) tested in the dedicated simulation study. Each polygon represents one peach orchard. All eight simulations start at year 1 from a unique introduction patch with 25% initial prevalence and spread is determined by the long-range kernel. Note that the final detected prevalence varies non-monotonically with detection sensitivity because the removal of detected trees reduces disease spread.
(TIFF)

S14 Fig Influence of detection sensitivity on the estimation of the long-range dispersal kernel. For each detection sensitivity, a single epidemic was simulated using the long-range kernel (black dashed curve). The posterior distributions of the estimated kernels (obtained from all MCMC chains with non-negligible mean posterior likelihood) are shown for three levels of prior information. Kernels are represented by their marginal probability density function *f*^1*D*^ (top row), and by their marginal cumulative distribution function *F*^1*D*^ with the distance from the source represented on the natural scale (middle row) or on the log_10_ scale (bottom row).
(TIFF)

S15 Fig Influence of detection sensitivity on the distance between simulated and estimated long-range dispersal kernels. For each of the 99 detection sensitivities, a single epidemic was simulated using the long-range kernel. For three levels of prior information, each bar represents a 95% credibility interval on the Kullback-Leibler distance (KLD) between simulated and estimated dispersal kernels (obtained from all MCMC chains with non-negligible mean posterior likelihood). The grey vertical lines correspond to the values of detection sensitivity used in S13 and S14 Figs.
(TIFF)

S16 Fig Estimated weights of the (BWME) dispersal kernel for the sharka epidemic. The posterior distribution of the weights (calculated with (Eq 11) for a mixture of 100 exponential kernels) is obtained for *κ* = 11 (i.e. the number of introduction patches maximising the Fisher information). The plotted posterior distribution of weights (as a function of the expected distance of each kernel) was obtained from 4000 MCMC samples. One line is plotted per sample.
(TIFF)

S17 Fig Estimated dispersal density for the sharka epidemic. The posterior distribution of the marginal probability density function, *f*^1*D*^, of the fitted dispersal kernel, obtained for *κ* = 11 (i.e. the number of introduction patches maximising the Fisher information). The plotted posterior distributions were obtained from 4000 MCMC samples. One line is plotted per sample.
(TIFF)

S1 Texts (A) Probabilistic framework for statistical inference, (B) prior distributions, (C) Markov chain Monte Carlo, and (D) model selection for *κ*. (PDF)
