# Correction: Estimation of the dispersal distances of an aphid-borne virus in a patchy landscape

**DOI:** 10.1371/journal.pcbi.1009315

**Published:** 2021-08-10

**Authors:** David R. J. Pleydell, Samuel Soubeyrand, Sylvie Dallot, Gérard Labonne, Joël Chadœuf, Emmanuel Jacquot, Gaël Thébaud

Fig 6 is incorrect. The authors have provided a corrected version here.

**Fig 6:**
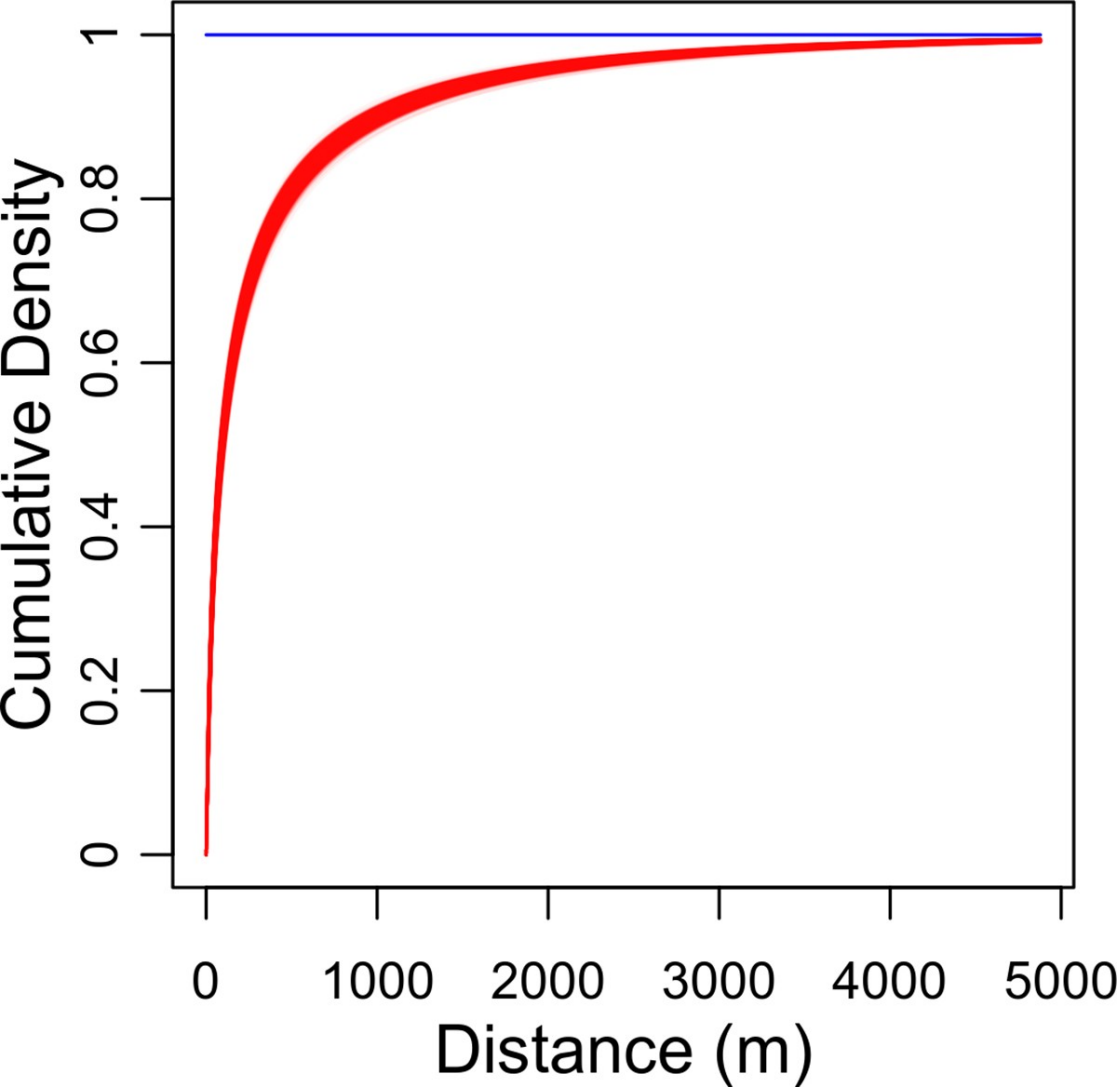
Estimated dispersal kernel for the sharka epidemic. The posterior marginal cumulative distribution function, *F*^1*D*^, of the fitted dispersal kernel, obtained for *κ* = 11 (i.e. the number of introduction patches maximising the Fisher information). The plotted posterior distribution was obtained from 4000 MCMC samples. One line is plotted per sample.
