# Supplementary figures and images for: Estimation of the dispersal distances of an aphid-borne virus in a patchy landscape
Source: PLoS Comput Biol. 2018 Apr 30;14(4):e1006085. doi: 10.1371/journal.pcbi.1006085 (PMC5945227; doi:10.1371/journal.pcbi.1006085)

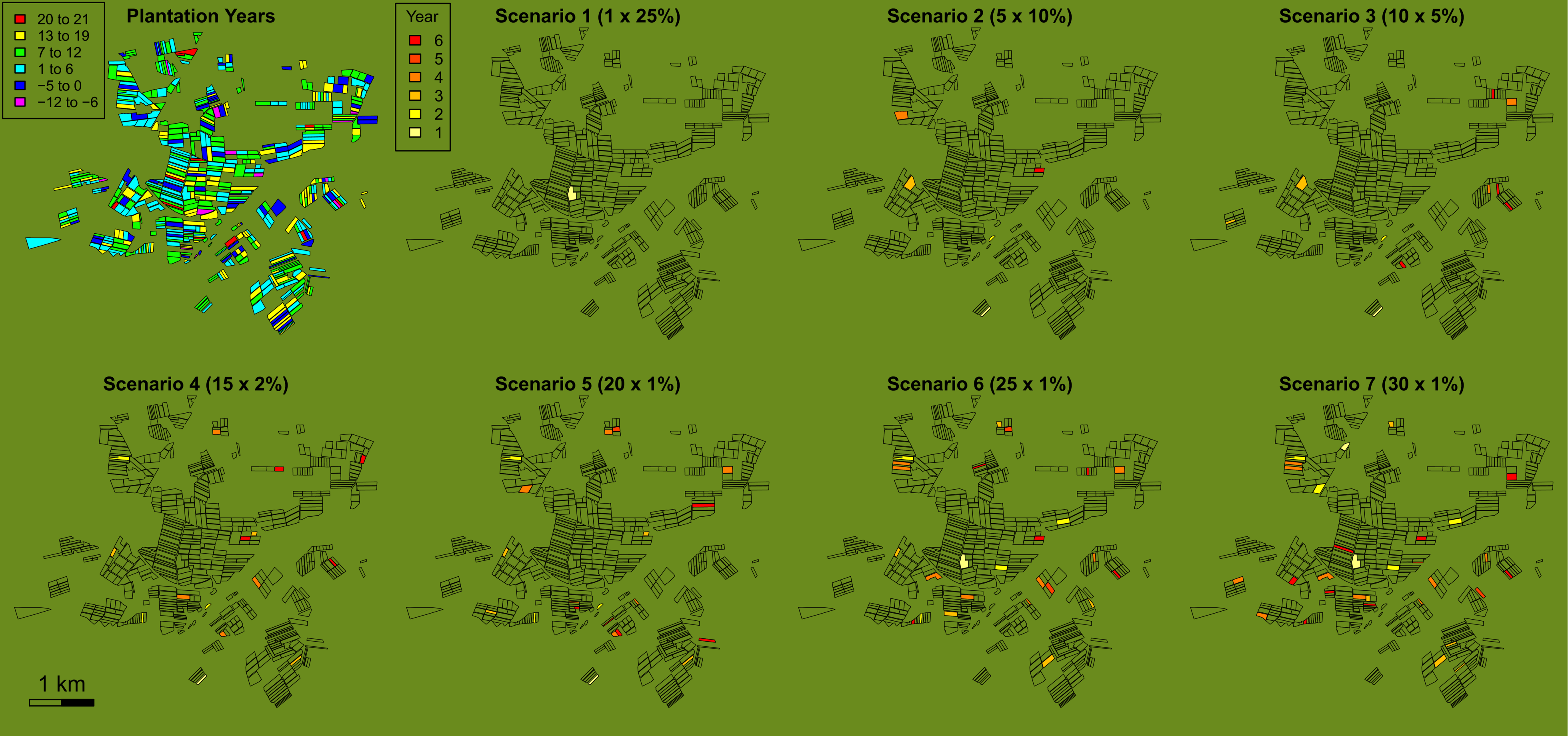

Supplement: S1 Fig — The first map (top left) represents the randomisation of the first planting years of the 553 patches. These years were sampled without replacement from their empirical distribution. The other maps show the location and planting year of each introduction patch in the seven introduction scenarios. The number of introduction patches and their initial prevalence are indicated for each introduction scenario. Note the greater landscape connectivity in the central area. (TIFF) [file pcbi.1006085.s001.tiff]

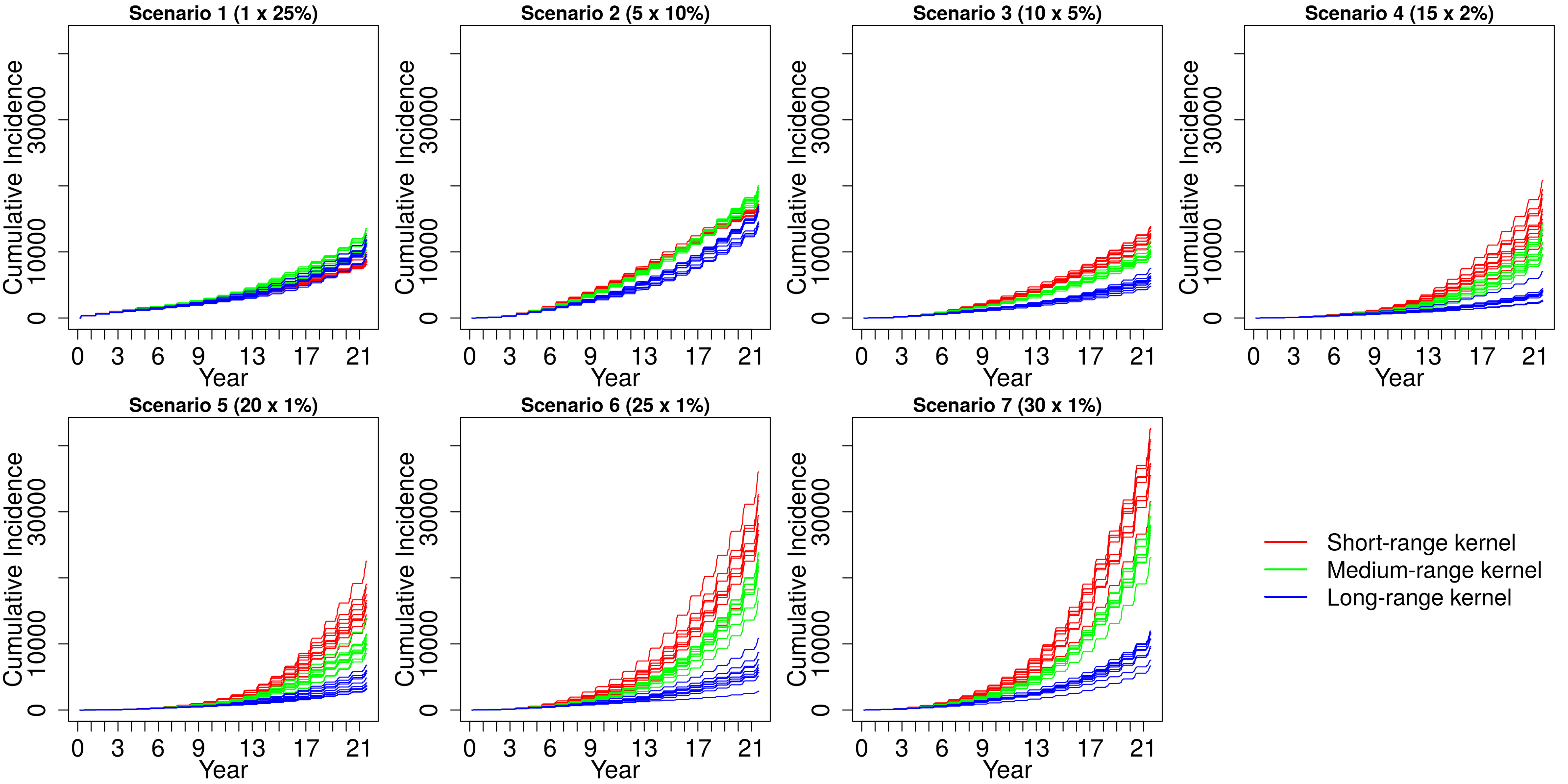

Supplement: S2 Fig — For each introduction scenario, the number of introduction patches and the corresponding initial disease prevalence are mentionned above the graph. The three tested kernels are represented by different colours. For each combination of kernel and introduction scenarios, 10 independant simulated epidemics are shown. (TIFF) [file pcbi.1006085.s002.tiff]

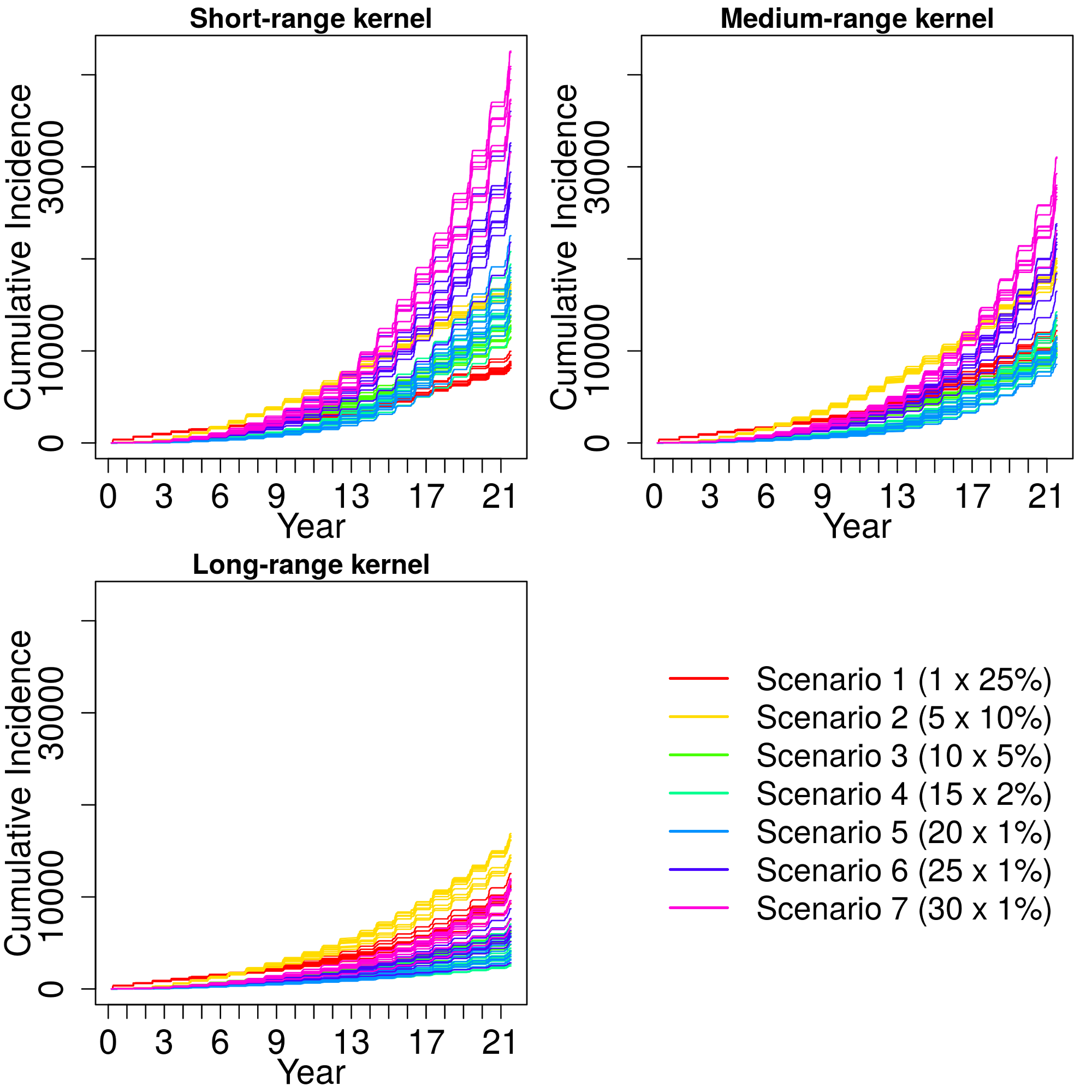

Supplement: S3 Fig — For each kernel, the seven tested introduction scenarios are represented by different colours. For each combination of kernel and introduction scenarios, 10 independant simulated epidemics are shown. (TIFF) [file pcbi.1006085.s003.tiff]

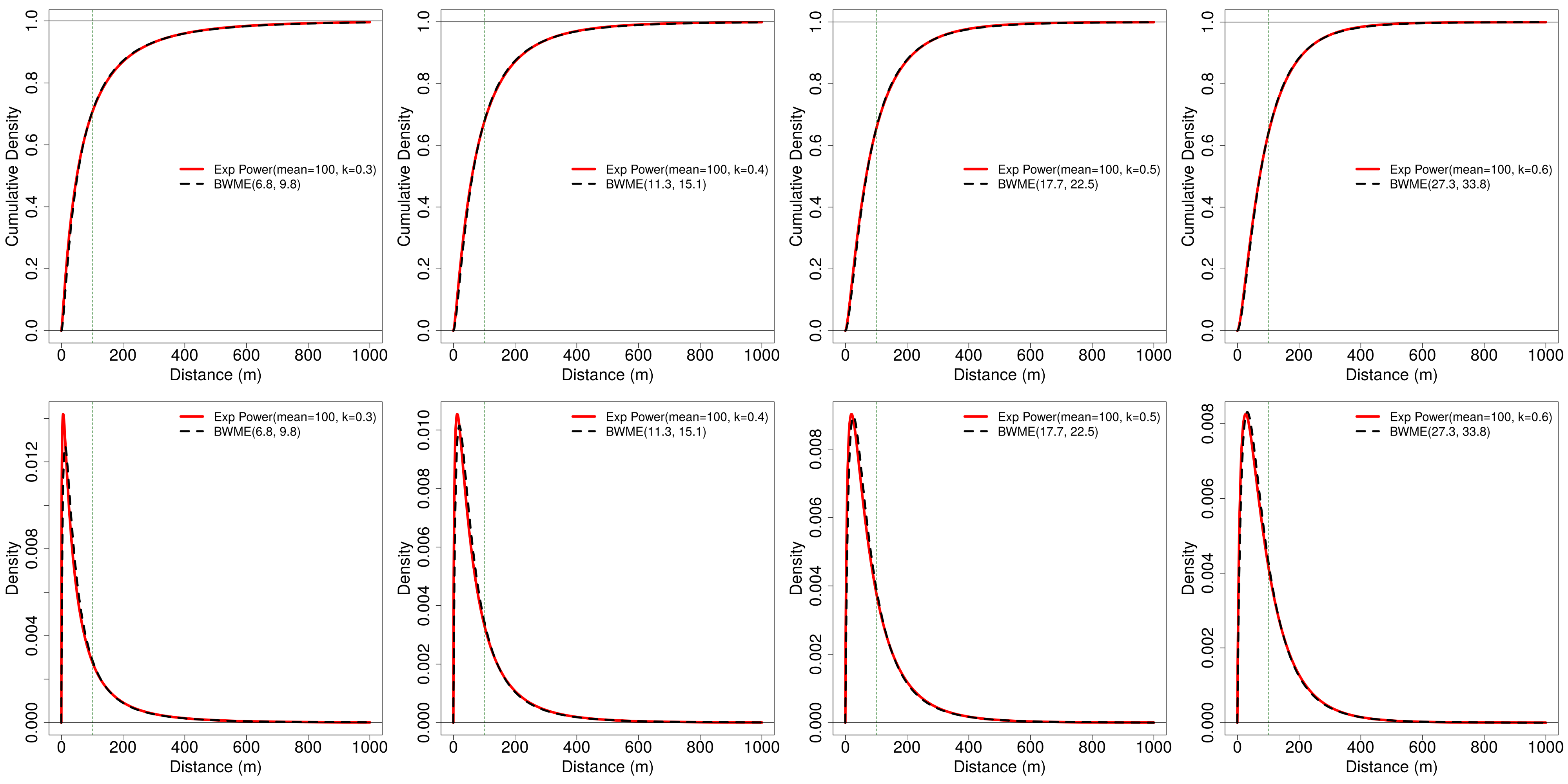

Supplement: S4 Fig — The kernels corresponding to 4 values of the shape parameter are represented by their cumulative distribution function F1D (top) and the associated probability density function f1D (bottom) of the distance travelled. Green dashed line: mean distance travelled. (TIFF) [file pcbi.1006085.s004.tiff]

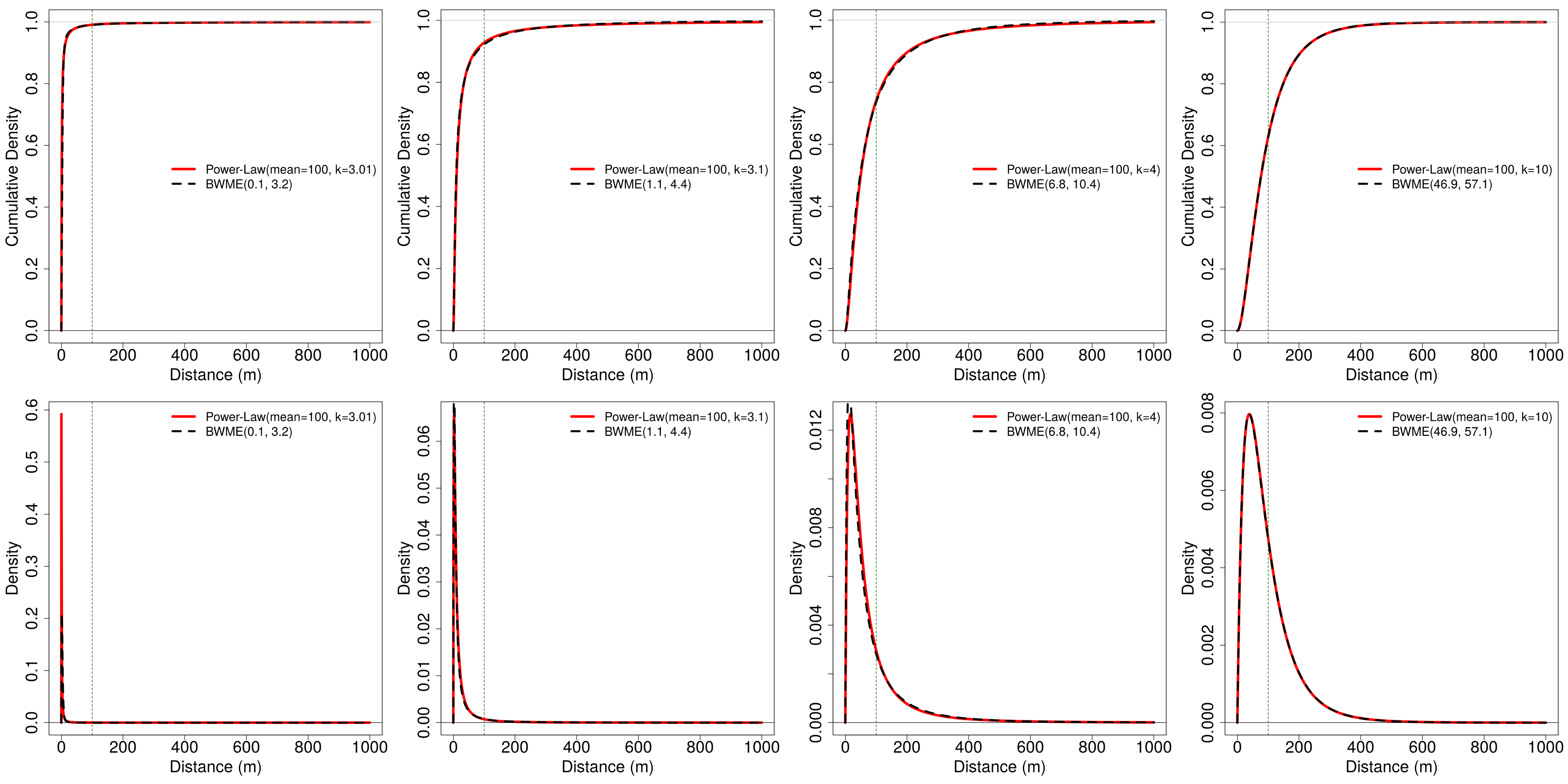

Supplement: S5 Fig — The kernels corresponding to 4 values of the shape parameter are represented by their cumulative distribution function F1D (top) and the associated probability density function f1D (bottom) of the distance travelled. Green dashed line: mean distance travelled. (TIFF) [file pcbi.1006085.s005.tiff]

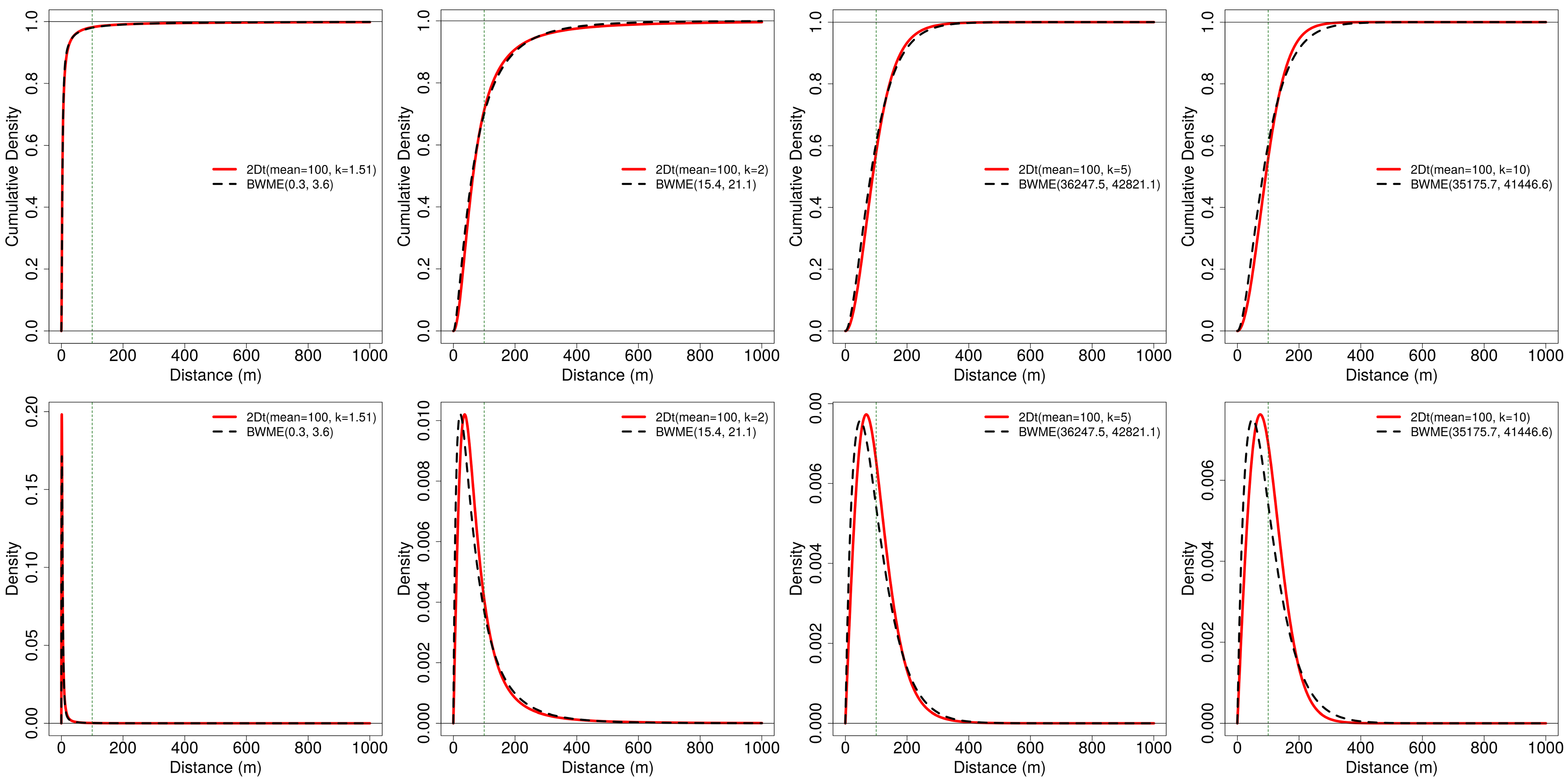

Supplement: S6 Fig — The kernels corresponding to 4 values of the shape parameter are represented by their cumulative distribution function F1D (top) and the associated probability density function f1D (bottom) of the distance travelled. Green dashed line: mean distance travelled. (TIFF) [file pcbi.1006085.s006.tiff]

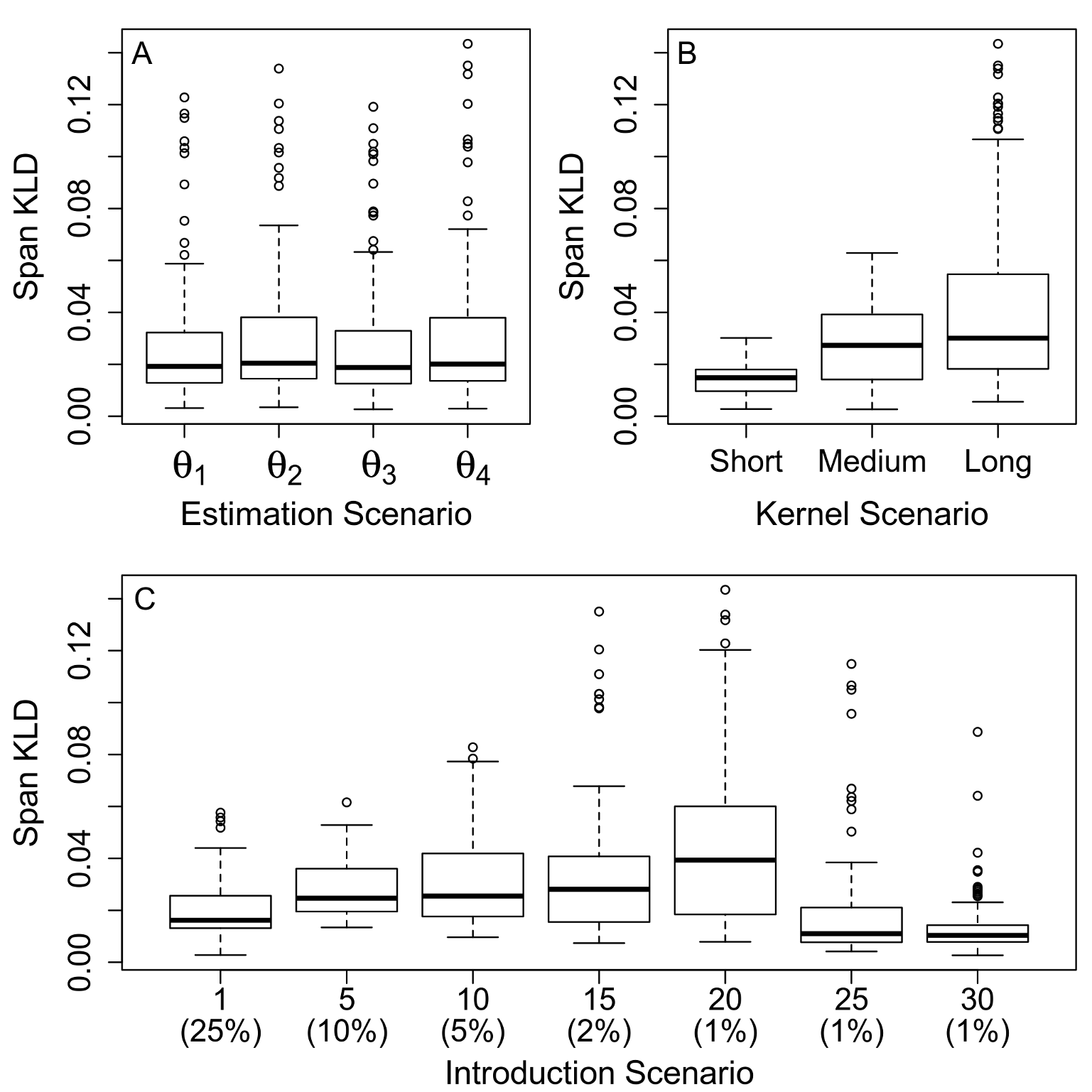

Supplement: S7 Fig — Impact of (A) estimation scenario, (B) kernel range, and (C) disease introduction scenario [number of introduction patches (with initial disease prevalence)] on the precision of estimated dispersal kernels. Precision is measured by the span of the 95% credibility interval of Kullback-Leibler distances (Span KLD) between simulated and estimated dispersal kernels. Each panel consists of 840 points, which correspond to 10 epidemics × 7 disease introduction scenarios × 3 dispersal kernels × 4 parameter estimation schemes. (TIFF) [file pcbi.1006085.s007.tiff]

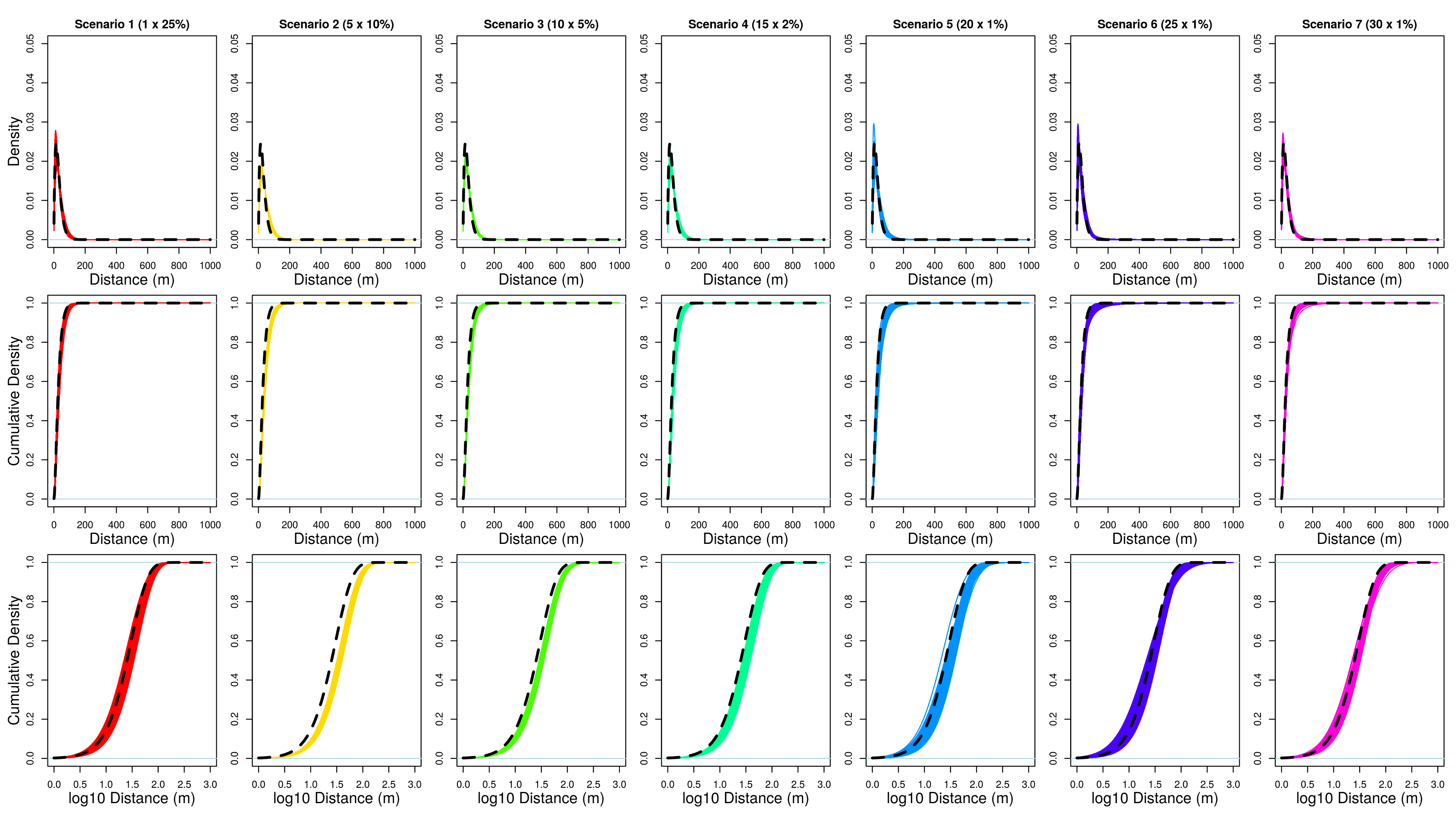

Supplement: S8 Fig — For each introduction scenario, 10 epidemics were simulated with a short-range kernel (black dashed curve), and 10 MCMC chains were run per simulated epidemic. The posterior distributions of the kernel obtained under the most exhaustive estimation scheme (Θ4) are represented for all chains with non-negligible mean posterior likelihood. The proportion of MCMC chains with negligible mean posterior likelihood (mean proportion: 10%) increases quadratically with the number of source orchards. Kernels are represented by their marginal probability density function f1D (top row), and by their marginal cumulative distribution function F1D with the distance from the source represented on the natural scale (middle row) or on the log10 scale (bottom row). (TIFF) [file pcbi.1006085.s008.tiff]

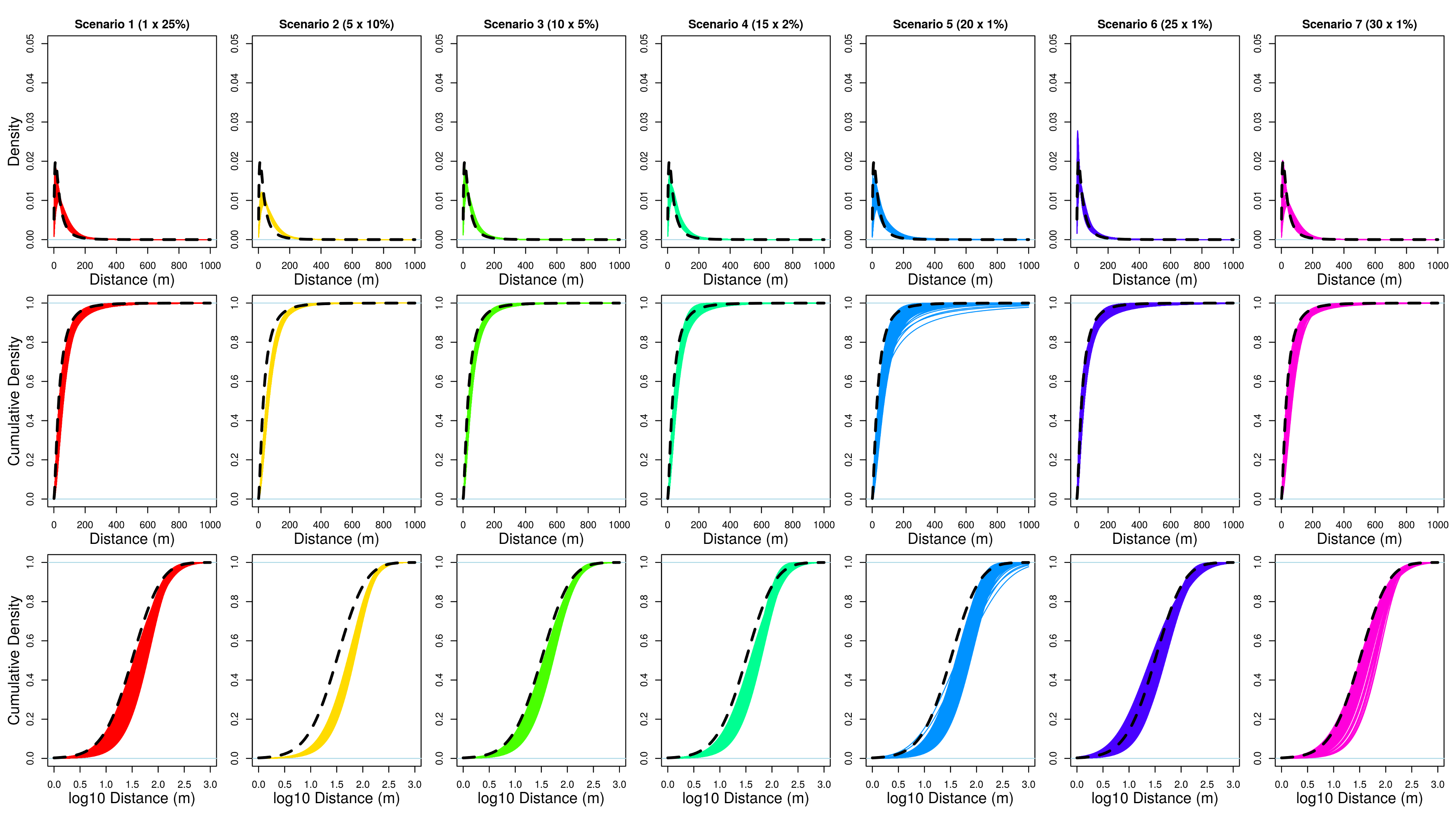

Supplement: S9 Fig — For each introduction scenario, 10 epidemics were simulated with a medium-range kernel (black dashed curve), and 10 MCMC chains were run per simulated epidemic. The posterior distributions of the kernel obtained under the most exhaustive estimation scheme (Θ4) are represented for all chains with non-negligible mean posterior likelihood. The proportion of MCMC chains with negligible mean posterior likelihood varies among introduction scenarios, with a mean proportion of 2.6%. Kernels are represented by their marginal probability density function f1D (top row), and by their marginal cumulative distribution function F1D with the distance from the source represented on the natural scale (middle row) or on the log10 scale (bottom row). (TIFF) [file pcbi.1006085.s009.tiff]

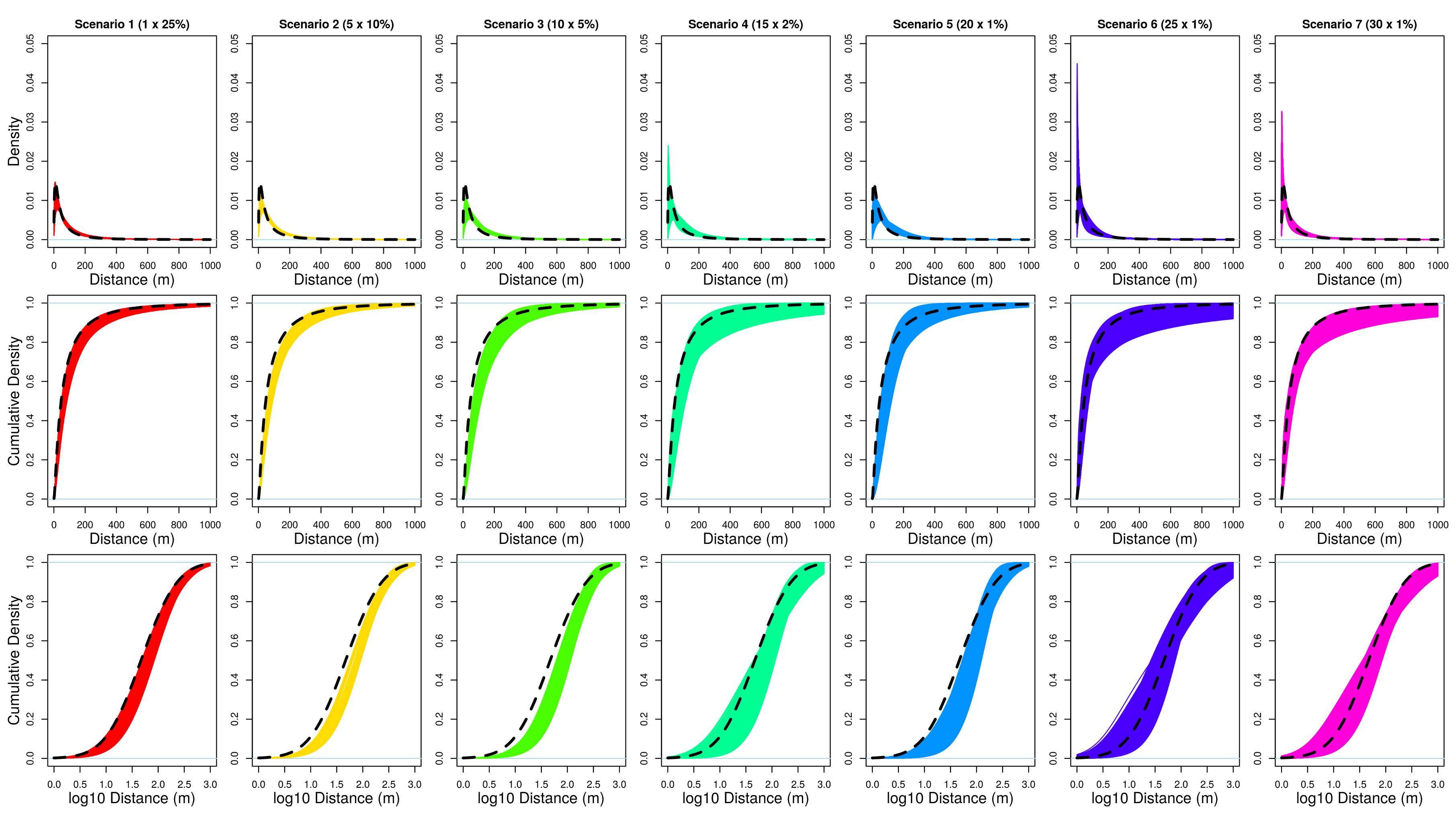

Supplement: S10 Fig — For each introduction scenario, 10 epidemics were simulated with a long-range kernel (black dashed curve), and 10 MCMC chains were run per simulated epidemic. The posterior distributions of the kernel obtained under the most exhaustive estimation scheme (Θ4) are represented for all chains with non-negligible mean posterior likelihood. The proportion of MCMC chains with negligible mean posterior likelihood is low (mean proportion: 0.4%) for all the introduction scenarios. Kernels are represented by their marginal probability density function f1D (top row), and by their marginal cumulative distribution function F1D with the distance from the source represented on the natural scale (middle row) or on the log10 scale (bottom row). (TIFF) [file pcbi.1006085.s010.tiff]

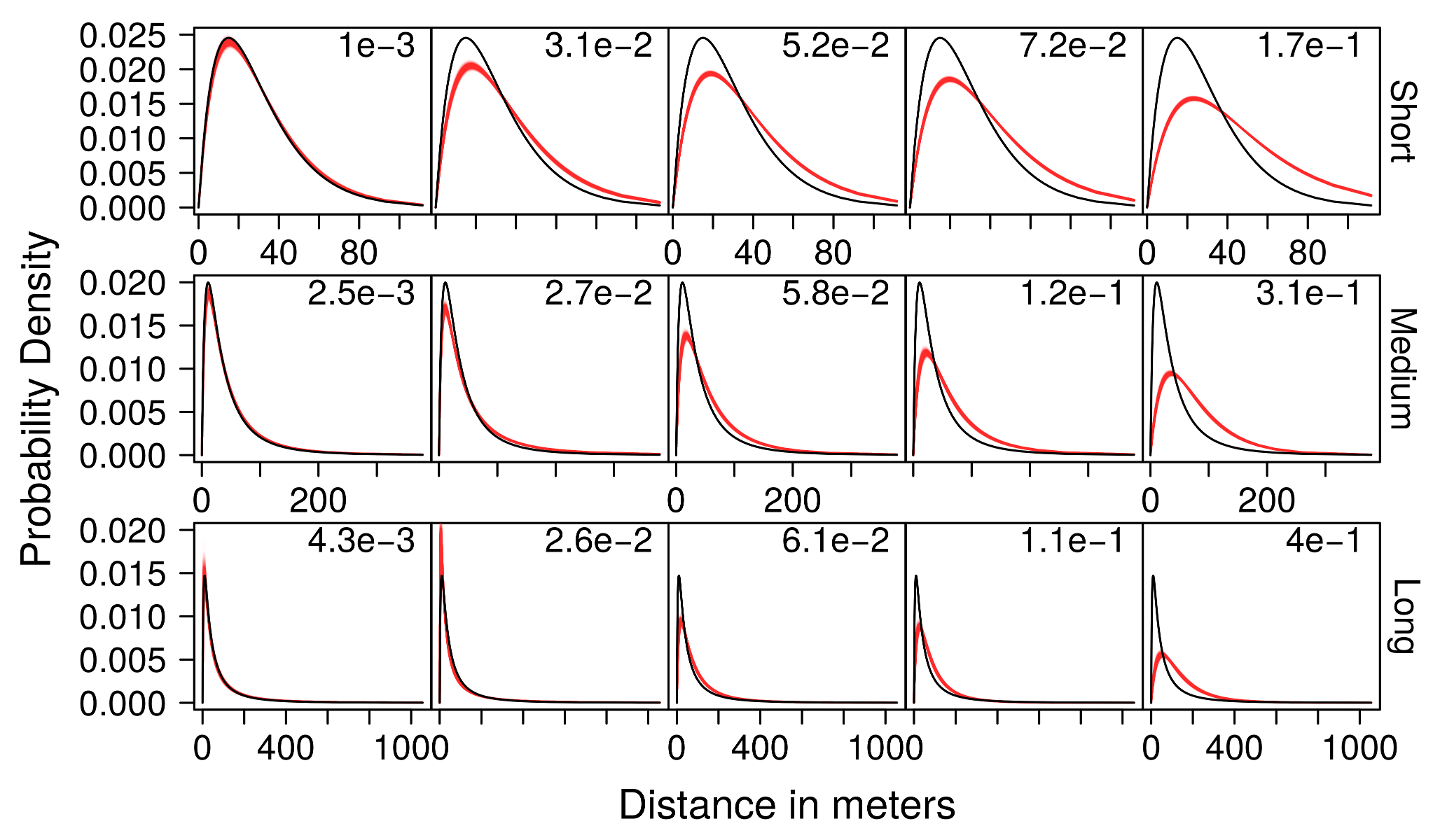

Supplement: S11 Fig — From left to right: kernels with the minimum, lower quartile, median, upper quartile and maximum Kullback-Leibler (KL) distances (posterior mean), for all chains with non-negligible mean posterior likelihood. Estimations (red) under the most exhaustive scheme (Θ4) are based on simulated epidemics with short-, medium- and long-range kernels (from top to bottom; black). Kernels are represented by their marginal probability density function f1D. The mean KL distance is indicated for each estimation. (TIFF) [file pcbi.1006085.s011.tiff]

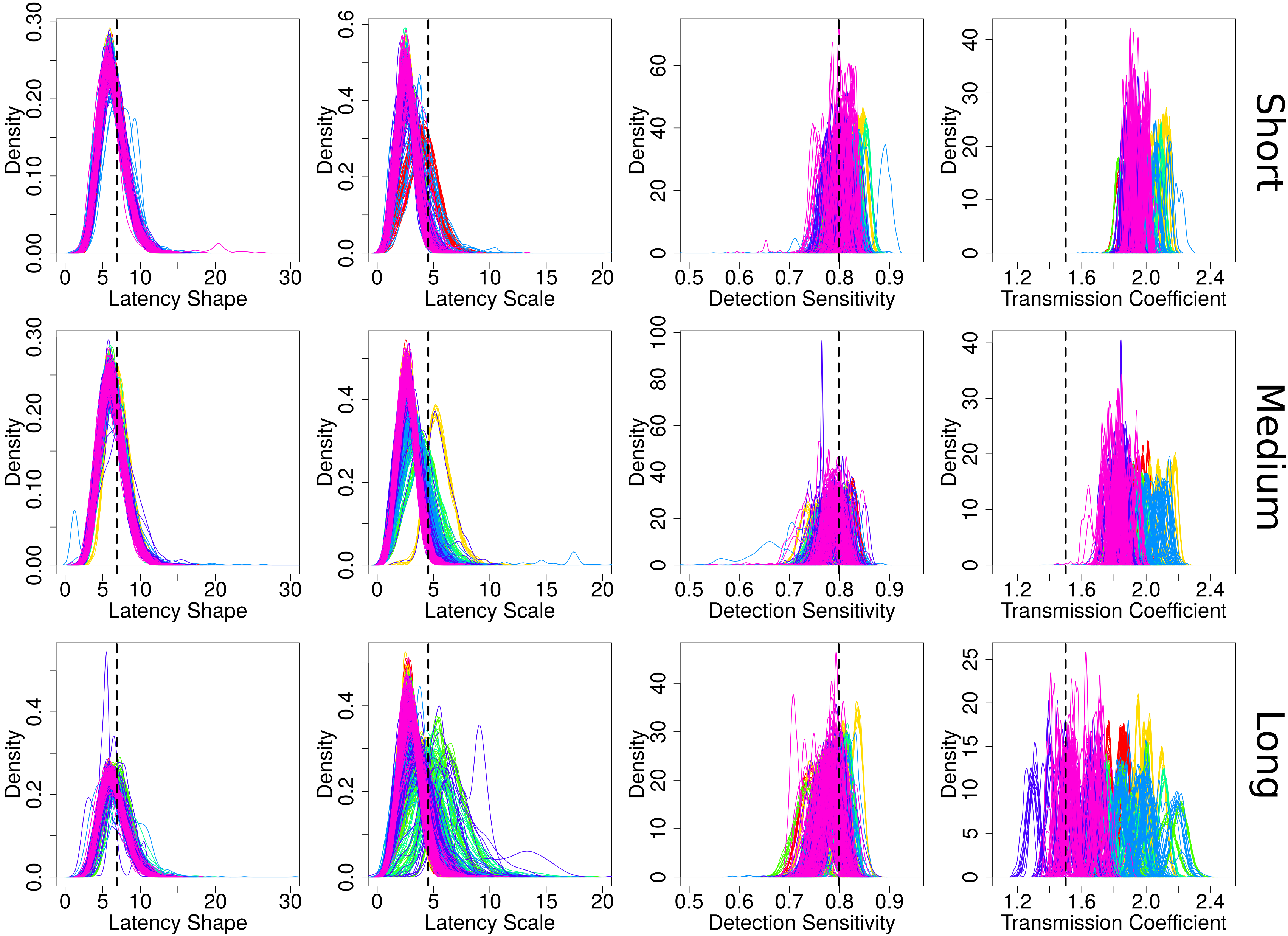

Supplement: S12 Fig — For each combination of short-, medium- and long-range kernels (from top to bottom) and introduction scenarios (colour-coded as in S3, S8, S9 and S10 Figs), 10 epidemics were simulated and 10 MCMC chains were run per simulated epidemic. The curves represent the posterior distribution of the parameters obtained under the most exhaustive estimation scheme (Θ4) for all chains with non-negligible mean posterior likelihood. Dashed lines: parameter values used in the simulations. (TIFF) [file pcbi.1006085.s012.tiff]

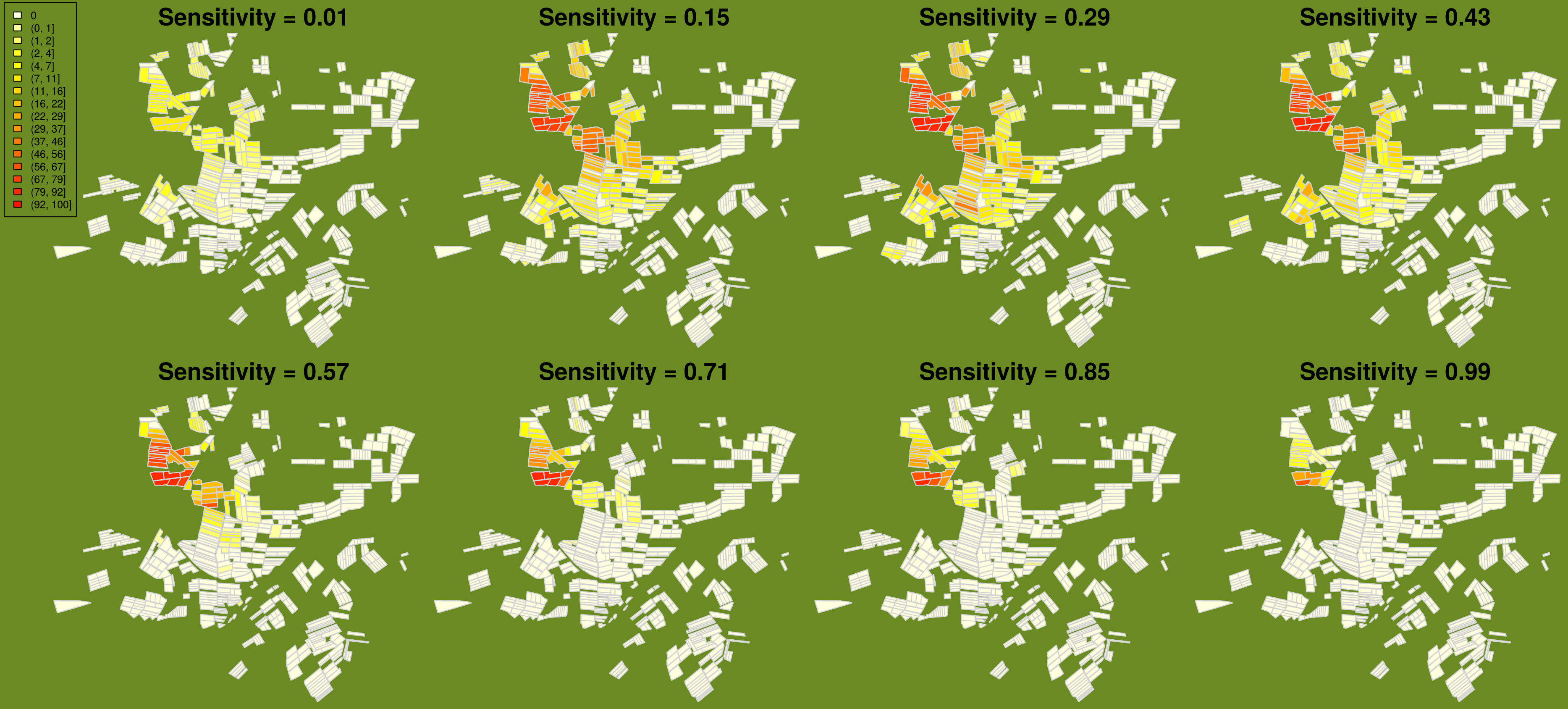

Supplement: S13 Fig — Each polygon represents one peach orchard. All eight simulations start at year 1 from a unique introduction patch with 25% initial prevalence and spread is determined by the long-range kernel. Note that the final detected prevalence varies non-monotonically with detection sensitivity because the removal of detected trees reduces disease spread. (TIFF) [file pcbi.1006085.s013.tiff]

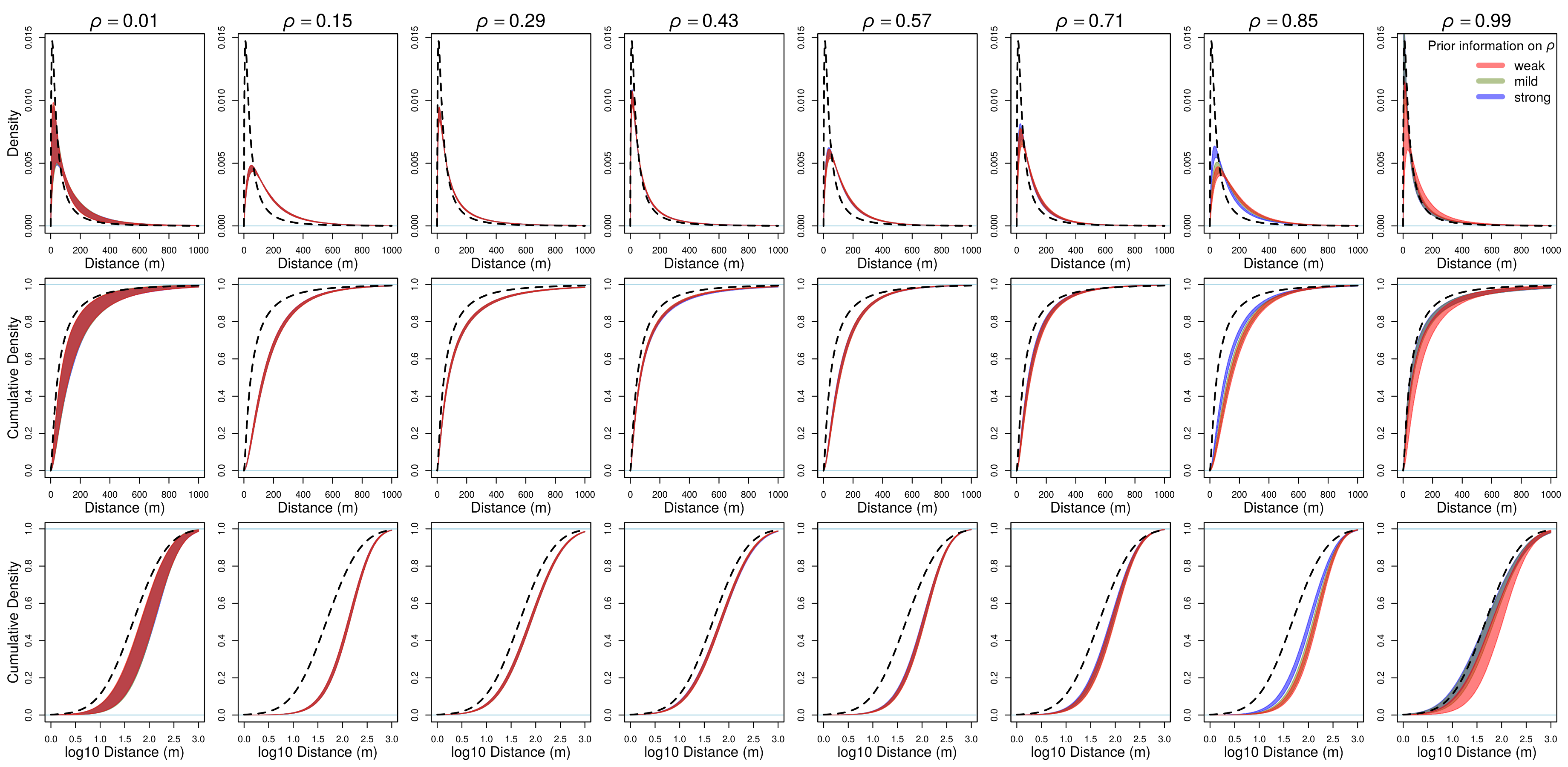

Supplement: S14 Fig — For each detection sensitivity, a single epidemic was simulated using the long-range kernel (black dashed curve). The posterior distributions of the estimated kernels (obtained from all MCMC chains with non-negligible mean posterior likelihood) are shown for three levels of prior information. Kernels are represented by their marginal probability density function f1D (top row), and by their marginal cumulative distribution function F1D with the distance from the source represented on the natural scale (middle row) or on the log10 scale (bottom row). (TIFF) [file pcbi.1006085.s014.tiff]

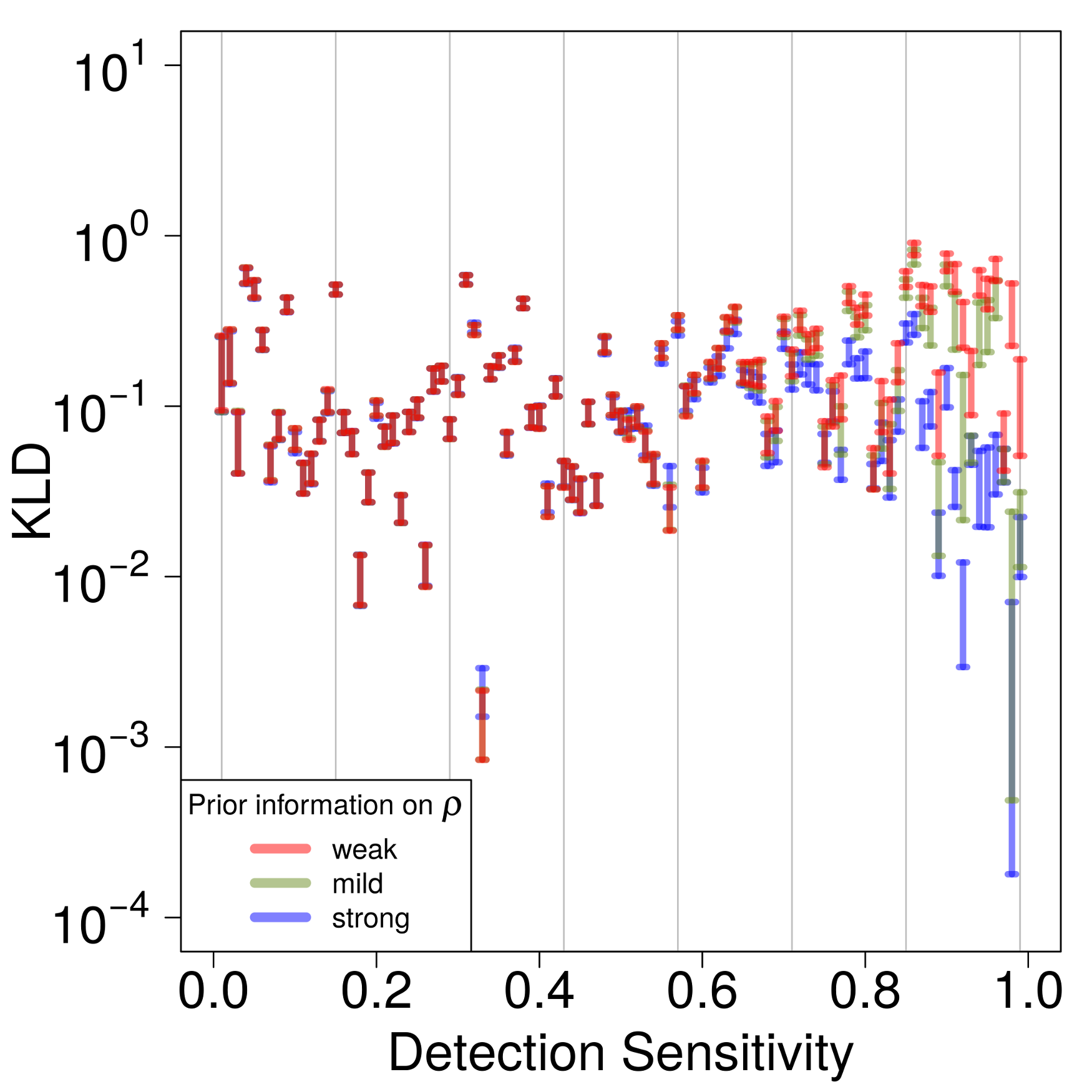

Supplement: S15 Fig — For each of the 99 detection sensitivities, a single epidemic was simulated using the long-range kernel. For three levels of prior information, each bar represents a 95% credibility interval on the Kullback-Leibler distance (KLD) between simulated and estimated dispersal kernels (obtained from all MCMC chains with non-negligible mean posterior likelihood). The grey vertical lines correspond to the values of detection sensitivity used in S13 and S14 Figs. (TIFF) [file pcbi.1006085.s015.tiff]

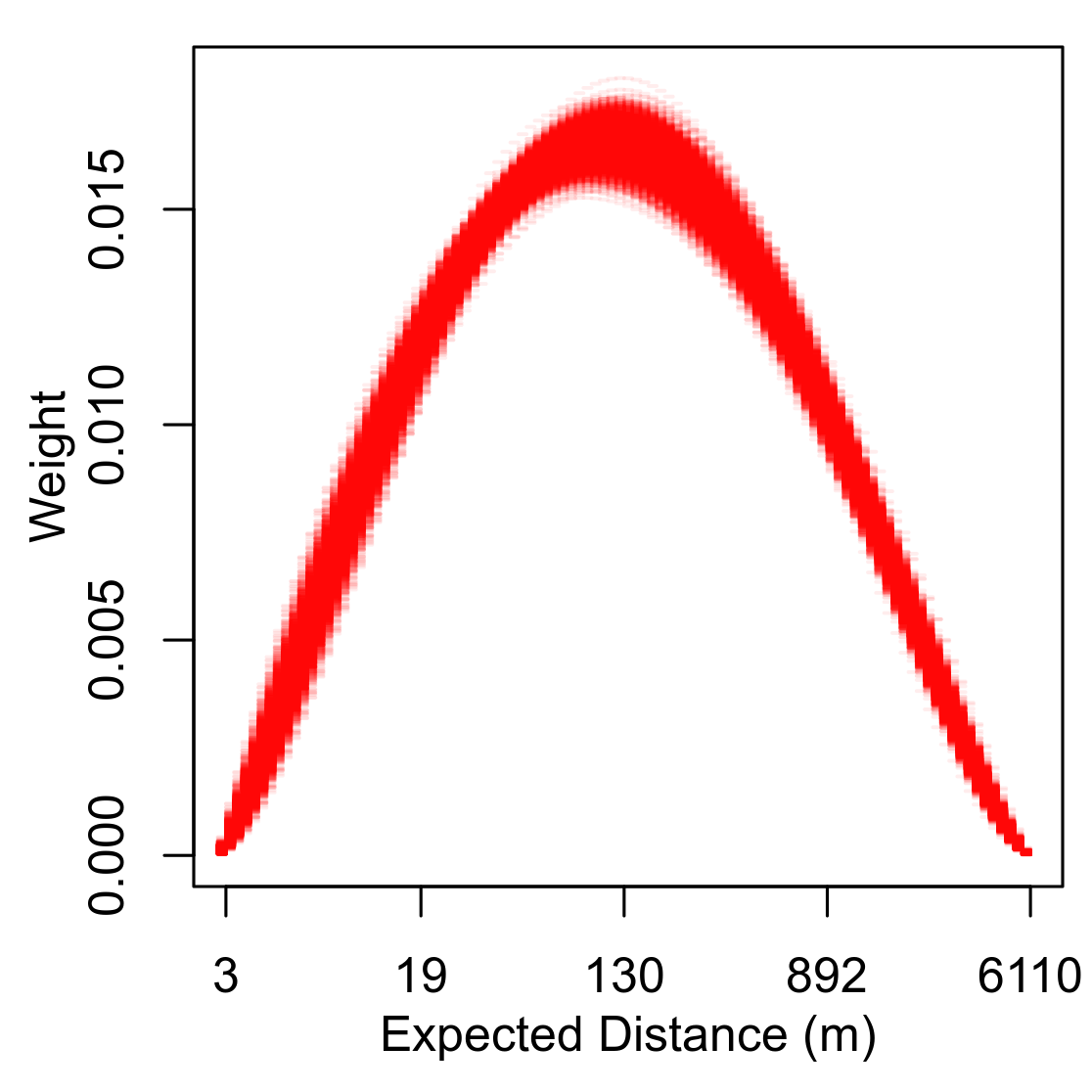

Supplement: S16 Fig — The posterior distribution of the weights (calculated with (Eq 11) for a mixture of 100 exponential kernels) is obtained for κ = 11 (i.e. the number of introduction patches maximising the Fisher information). The plotted posterior distribution of weights (as a function of the expected distance of each kernel) was obtained from 4000 MCMC samples. One line is plotted per sample. (TIFF) [file pcbi.1006085.s016.tiff]

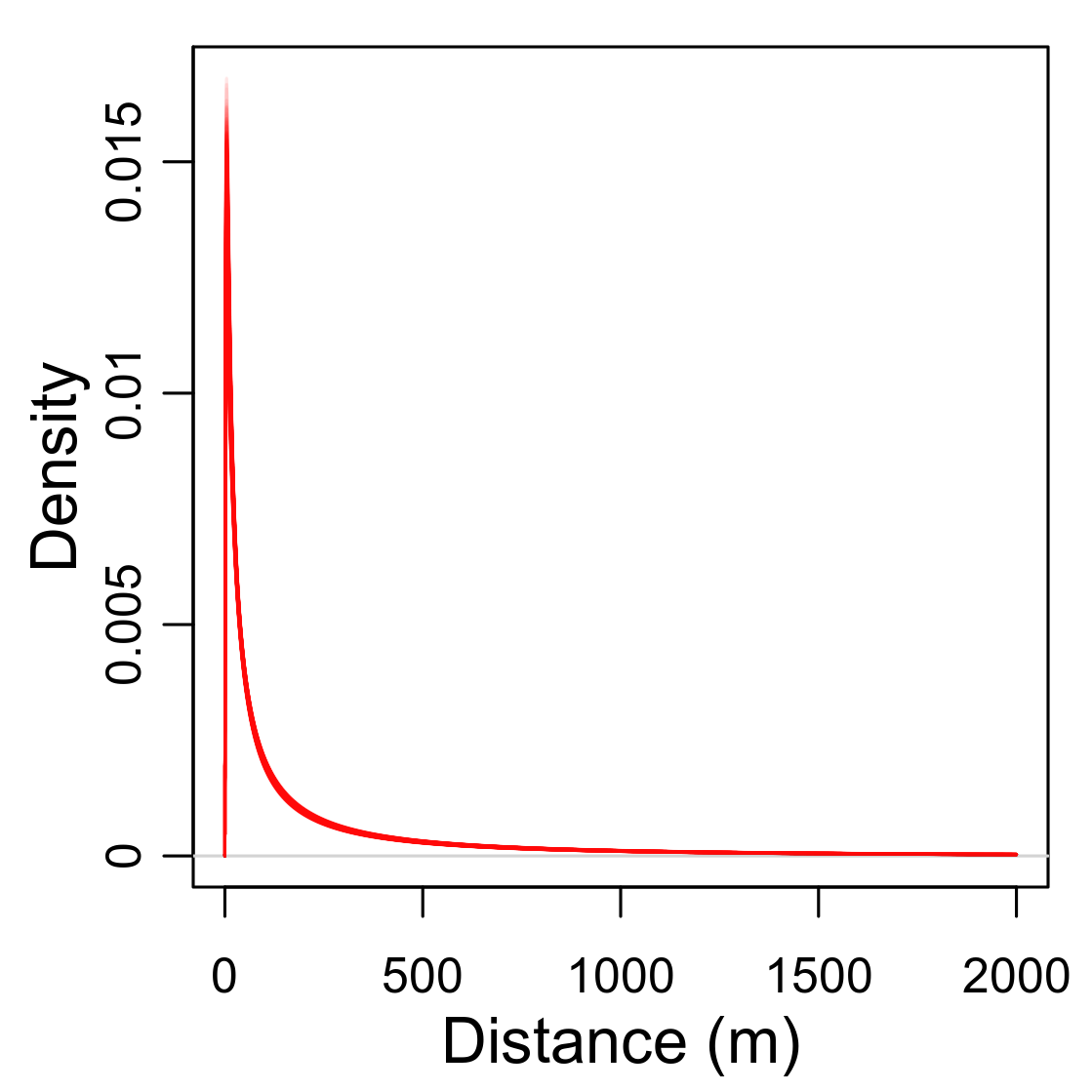

Supplement: S17 Fig — The posterior distribution of the marginal probability density function, f1D, of the fitted dispersal kernel, obtained for κ = 11 (i.e. the number of introduction patches maximising the Fisher information). The plotted posterior distributions were obtained from 4000 MCMC samples. One line is plotted per sample. (TIFF) [file pcbi.1006085.s017.tiff]
