# Supplementary material for: Estimation of the dispersal distances of an aphid-borne virus in a patchy landscape
Source: PLoS Comput Biol. 2018 Apr 30;14(4):e1006085. doi: 10.1371/journal.pcbi.1006085 (PMC5945227; doi:10.1371/journal.pcbi.1006085)
Supplement: S1 Texts — (PDF) [file pcbi.1006085.s018.pdf]

Text A: Probabilistic Framework for Statistical Inference

Epidemic reconstruction is performed in a Bayesian framework, using one time step per year for estimation. For a fixed number κ of introduction patches, estimation is based on the posterior distribution of parameter set Θ given observed data Y:

$$f(\Theta|Y,\kappa) = \frac{\widehat{l}(Y|\Theta)f(\Theta|\kappa)}{\int \widehat{l}(Y|\Theta)f(\Theta|\kappa)d\Theta}, \tag{S1}$$

where $f(\Theta|Y,\kappa)$ is the joint probability density of parameters Θ given data Y and κ, $f(\Theta|\kappa)$ is the prior density of Θ given κ, and $\widehat{l}(Y|\Theta)$ is the pseudo-likelihood of Y given Θ. We call $\widehat{l}$ a pseudo-likelihood since it exploits certain approximations to simplify computation (see below). Here, Y and Θ simply represent "all data" and "all parameters" respectively; a more detailed presentation is given below (Texts B and C in S1 Texts).

In what follows, $T_{E,i}$, $T_{H,i}$, $T_{D,i}$ and $T_{R,i}$ are the dates at which a given (randomly selected) individual from patch i makes the transition to compartments E, H, D and R respectively. For a given individual of patch i, $T_{D,i}$ is the date at which symptoms were observed (for the first time), so $T_{D,i}$ corresponds either to one of the K_i inspection dates in patch i, or is right-censored. Since our model is designed for the analysis of patch-level data, we assume that $p(T_{D,i} = t_{i,k})$ (the probability for a tree to be detected at the kth inspection date in patch i) is homogeneous within patch i at any given time. Let N_i be the initial number of trees planted in patch i, $D^+_{i,k}$ be the number of newly detected infectious individuals (among symptomatic individuals) at each inspection k, and D^-_i be the number of (uninfected or infected) individuals upon which symptoms were not detected in any of the K_i inspections. Based on Eq (12), and noting that $D^-_i = N_i - \sum_{k=1}^{K_i} D^+_{i,k}$, the pseudo-likelihood of the observed data is:

$$\widehat{l}(Y|\Theta) = \prod_i M_i \left[\left(1 - \sum_{k=1}^{K_i} p(T_{D,i} = t_{i,k})\right)^{N_i - \sum_{k=1}^{K_i} D^+_{i,k}} \prod_{k=1}^{K_i} p(T_{D,i} = t_{i,k})^{D^+_{i,k}} \right], \tag{S2}$$

where the multinomial coefficient for patch i, M_i, is constant with respect to Θ. We now address each transition in the $SEHDR$ framework to indicate how spatial structure and transition-time censoring are accounted for when constructing the probabilities in Eq (S2).

T_E: Transmission

Let r denote the time step of the model. The probability that a given individual in patch i became infected in time interval $(t_{r-1}, t_r]$ is modelled as:

$$p(t_{r-1} < T_{E,i} \leq t_r) = p(t_{r-1} < T_{E,i} \leq t_r | t_{r-1} < T_{E,i}) \times p(t_{r-1} < T_{E,i}). \tag{S3}$$

Let $T_{0,i}$ denote the time at which patch i is initialised (i.e. the beginning of the first year of budbreak in patch i). Let Z_i be a Boolean random variable indicating whether (Z_i=1) or not (Z_i=0) patch i is an introduction patch (with initial prevalence p_i). The probability that an individual is still susceptible at t_{r-1} is:

$$p(t_{r-1} < T_{E,i}) = p(T_{0,i} < T_{E,i}|Z_i) \prod_{r'=1}^{r-1} p(t_{r'} < T_{E,i} | t_{r'-1} < T_{E,i}), \tag{S4}$$

where $p(T_{0,i} < T_{E,i}|Z_i) = 1 - p_i Z_i$, and where the conditional probability of remaining susceptible throughout a time step is:

$$p(t_r < T_{E,i} | t_{r-1} < T_{E,i}) = 1 - p(t_{r-1} < T_{E,i} \leq t_r | t_{r-1} < T_{E,i}). \tag{S5}$$

Assuming independence between infection events within each time interval $(t_{r-1}, t_r]$, the probability that at least one infection event affected an individual within $(t_{r-1}, t_r]$ is:

$$p(t_{r-1} < T_{E,i} \leq t_r | t_{r-1} < T_{E,i}) = 1 - e^{-\lambda_{i,t_r}}, \tag{S6}$$

and thus:

$$p(t_{r-1} < T_{E,i} \leq t_r) = (1 - p_i Z_i)(1 - e^{-\lambda_{i,t_r}}) e^{-\sum_{r'=1}^{r-1} \lambda_{i,t_{r'}}}, \tag{S7}$$

where the force of infection λ_{i,t_r} is defined (main text, Eq (5)) as the expected number of infection events affecting a given individual of patch i in $(t_{r-1}, t_r]$. Note that $I_{i,t_{r-1}}$ and $R_{i,t_{r-1}}$ in Eq (5) are unknown. Instead of using data augmentation to directly incorporate hundreds of unobserved infection times within the MCMC algorithm, we used a pseudo-likelihood [1] where these variables are replaced by their expected values:

$$E[I_{i,t_{r-1}}] = N_i \times \left[1 - p(t_{r-1} < T_{H,i}) - p(T_{R,i} \leq t_{r-1})\right] \text{ and} \tag{S8}$$

$$E[R_{i,t_{r-1}}] = N_i \times p(T_{R,i} \leq t_{r-1}). \tag{S9}$$

The term in square brackets in Eq (S8) gives the expected proportion of individuals in state H or D at time t_{r-1}. Similarly, the probability term in Eq (S9) gives the expected proportion of individuals in state R at time t_{r-1}. The terms $p(t_{r-1} < T_{H,i})$ and $p(T_{R,i} \leq t_{r-1})$ are derived below (in Eqs (S12) and (S17), respectively).

T_H: the end of the latent period

The marginal probability for an individual to be in compartment S or E at time t_r is found by integrating over all possibilities of the unknown infection time $T_{E,i}$ as follows:

$$\begin{aligned} p(t_r < T_{H,i}) =& p(t_r < T_{E,i}) + p(t_r < T_{H,i}|T_{E,i} \leq T_{0,i}) \times p(T_{E,i} \leq T_{0,i}) + \\ & \sum_{r'=1}^{r} \Big[p(t_r < T_{H,i}|t_{r'-1} < T_{E,i} \leq t_{r'}) \times p(t_{r'-1} < T_{E,i} \leq t_{r'})\Big]. \end{aligned} \tag{S10}$$

The conditional probability for an individual to be still in the exposed state at t_r given that it became infected in a previous time interval $(t_{r'-1}, t_{r'}]$ is modelled as:

$$p(t_r < T_{H,i}|t_{r'-1} < T_{E,i} \leq t_{r'}) = 1 - F_{\text{Tr,lat}}(t_r - t_{r'}), \tag{S11}$$

where $F_{\text{Tr,lat}}$ is the cumulative distribution function of the left-truncated gamma distribution with shape θ_1 and scale θ_2. In the case of PPV-M the truncation is used to represent a minimal latent period of one winter, and we assume $p(t_r < T_{H,i}|T_{E,i} \leq T_{0,i}) = 0$. Thus:

$$p(t_r < T_{H,i}) = p(t_r < T_{E,i}) + \sum_{r'=1}^{r} \Big(\left[1 - F_{\text{Tr,lat}}(t_r - t_{r'})\right] \times p(t_{r'-1} < T_{E,i} \leq t_{r'})\Big). \tag{S12}$$

T_D: Detection

At each inspection, infectious trees can be detected on the basis of sharka-specific symptoms that appear mainly on flowers and leaves when the latent period is over. Thus, we assume that only trees in state H can be detected (as infected). The probability that symptoms are detected for the first time on a given tree in patch i at inspection date $t_{i,k}$ is:

$$\begin{aligned} p(T_{D,i} = t_{i,k}) &= p(T_{D,i} = t_{i,k}, T_{D,i} > t_{i,k-1}, T_{H,i} \leq t_{i,k}) \\ &= p(T_{D,i} = t_{i,k}|T_{D,i} > t_{i,k-1}, T_{H,i} \leq t_{i,k}) \times p(T_{D,i} > t_{i,k-1}, T_{H,i} \leq t_{i,k}), \end{aligned} \tag{S13}$$

where the first term corresponds to the detection sensitivity ρ_{i,t_r}, and the second term is derived as follows:

$$\begin{aligned} p(T_{D,i} > t_{i,k-1}, T_{H,i} \leq t_{i,k}) &= p(T_{H,i} \leq t_{i,k}) - p(T_{D,i} \leq t_{i,k-1}, T_{H,i} \leq t_{i,k}) \\ &= p(T_{H,i} \leq t_{i,k}) - p(T_{D,i} \leq t_{i,k-1}) \times p(T_{H,i} \leq t_{i,k}|T_{D,i} \leq t_{i,k-1}). \end{aligned} \tag{S14}$$

Because $T_{H,i} \leq T_{D,i}$ for any given individual, the last term of Eq (S14) is equal to one. Thus, we obtain the relation:

$$p(T_{D,i} > t_{i,k-1}, T_{H,i} \leq t_{i,k}) = p(T_{H,i} \leq t_{i,k}) - \sum_{k'<k} p(T_{D,i} = t_{i,k'}). \tag{S15}$$

The probability $p(T_{H,i} \leq t_{i,k})$ of having passed into compartment H prior to, or at, $t_{i,k}$ is derived directly from Eq (S12). Note that when more than one inspection date falls within a single time step, the order of these inspections is preserved, i.e. Eqs (S13) and (S15) do not change and the probability of detecting an infectious tree for the first time at the second inspection date will be lower than at the first date.

T_R: Removal

Although removal dates for whole orchards were recorded, this was not the case for the removal of individual trees. We adopt a discrete-time survival model to account for this censoring in which detected infectious trees in patch i are removed each time step with a probability determined by the mean duration δ between detection and removal. Thus, the probability for a tree in patch i to be removed before time t_r is modelled as:

$$p(T_{R,i} < t_r) = \sum_{\{k:\, t_{i,k} < t_r\}} \Big[p(T_{R,i} < t_r | T_{D,i} = t_{i,k}) \times p(T_{D,i} = t_{i,k}) \Big] \quad \text{(S16)}$$

$$= \sum_{\{k:\, t_{i,k} < t_r\}} \bigg(\Big[1 - p(T_{R,i} > t_r | T_{R,i} > t_{r-1}, T_{D,i} = t_{i,k})^{\Delta(t_r - t_{i,k})} \Big] \times p(T_{D,i} = t_{i,k}) \bigg), \quad \text{(S17)}$$

where $\Delta(t_r - t_{i,k})$ is the number of time steps between t_r and the start of the time step containing inspection date $t_{i,k}$. Here, $p(T_{R,i} > t_r | T_{R,i} > t_{r-1}, T_{D,i} = t_{i,k})$ is the probability that an individual in state D at time t_{r-1} still is in state D by time t_r. In practice, the vast majority of detected trees are removed within the legal 10-day delay, and almost all detected trees are removed before the end of the growing season. However, since we use a 1-year time step for estimation, we assume $p(T_{R,i} > t_r | T_{R,i} > t_{r-1}, T_{D,i} = t_{i,k})$ is zero when t_r and $t_{i,k}$ belong to different civil years and is one otherwise.

Text B: Prior Distributions

The priors used throughout this work are:

$$\begin{aligned}
\mu &\sim \text{Uniform}(0, 1), \\
\sigma &\sim \text{Exponential}(\text{scale} = 10^3), \\
\theta_1 &\sim \text{Gamma}(15.5, 0.444), \\
\theta_2 &\sim \text{Gamma}(5.11, 0.888), \\
\log(\beta) &\sim \text{Uniform}(-\infty, \infty), \\
\rho &\sim \text{Beta}(559, 141), \\
Z_i &\sim \text{Bernoulli}(0.5), \\
p_i | Z_i = 1 &\sim \text{Uniform}(0, 1), \\
\widehat{X}^{\text{mis}}_{\text{pl},i} &\sim \text{Empirical}(\boldsymbol{X}_{\text{pl}}), \\
\widehat{X}^{\text{mis}}_{\text{in},i,k} | \mathcal{T}_{i,k} &\sim \text{Empirical}(\boldsymbol{X}_{\text{in}} | \mathcal{T}_{i,k}),
\end{aligned}$$

where: $\mu = \frac{s_1}{s_1+s_2}$ and $\sigma = s_1 + s_2$ are parameters of the BWME kernel (Eq 11); θ_1 and θ_2, respectively, are shape and scale parameters for the latent period model (Eq 2); β is the transmission coefficient (Eq 5); ρ is the detection sensitivity (Eq 3); Z_i is a variable indicating whether patch i is an introduction patch (Z_i=1) or not (Z_i=0); p_i, the introduction prevalence in patch i (Eqs S4 and S7), is set to zero when patch i is not an introduction patch; $\widehat{X}^{\text{mis}}_{\text{pl},i}$ are imputed values for missing planting dates which are assumed *a priori* to be distributed according to the empirical distribution of known planting dates $\boldsymbol{X}_{\text{pl}}$; $\widehat{X}^{\text{mis}}_{\text{in},i}$ are imputed values for missing inspection dates which are assumed *a priori* to be distributed according to the empirical distribution of known inspection dates $\boldsymbol{X}_{\text{in}}$ conditioned on inspection type data $\mathcal{T}_{i,k}$ indicating whether inspection k in patch i focused on symptoms on either flowers (i.e. early spring) or leaves (i.e. late spring). Based on field and laboratory observations by SD and GL, hyper-parameters for the latent period model are set using weeks as the temporal unit, with a prior mean and variance of 6.9 and 3.1 for θ_1, and 4.5 and 4.0 for θ_2 (which corresponds to θ_{exp}=31 and θ_{var}=435). Hyper-parameters for the prior sensitivity to detect infectious individuals are based on available field data and give a mode et 0.80 and a variance of 0.00023.

For the study of the impact of ρ on estimation, the prior distribution of detection sensitivity is defined as Beta(1+$\rho\omega$, 1+(1-ρ)ω), with ω=1, 100 or 10000 corresponding to weak, mild or strong prior knowledge, respectively. The mode of these priors matches the simulated value of ρ and the associated precision increases with ω (e.g. for ρ=0.8, the variance of the prior is equal to 0.06, 1.6×10^{-3}, and 1.6×10^{-6}, respectively).

Text C: Markov Chain Monte Carlo

Bayesian inference is based on Markov chain Monte Carlo (MCMC) approximation of the joint distribution:

$$f(\mu, \sigma, \beta, \theta_1, \theta_2, \rho, \boldsymbol{p}, \boldsymbol{Z}, \widehat{\boldsymbol{X}}_{\text{pl}}^{\text{mis}}, \widehat{\boldsymbol{X}}_{\text{in}}^{\text{mis}} \,|\, \boldsymbol{Y}, \boldsymbol{X}_{\text{pl}}, \boldsymbol{X}_{\text{in}}, \boldsymbol{\mathcal{T}}, \kappa), \tag{S18}$$

where: $f(\cdot|\cdot)$ represents the conditional probability density of a given subset of parameters; $\boldsymbol{Z}=\{Z_1, \ldots, Z_i, \ldots\}$ is the set of variables specifying whether each patch i is classified as an introduction patch (Z_i=1) or not (Z_i=0); $\boldsymbol{p}=\{p_1, \ldots, p_i, \ldots\}$ is the corresponding set of introduction prevalences; $\boldsymbol{Y}$ represents the data associated with each inspection, including the number of infectious individuals detected, for the first time, in each orchard at each inspection; $\boldsymbol{X}_{\text{pl}}$ and $\boldsymbol{X}_{\text{in}}$ are sets of known planting and inspection dates respectively; $\widehat{\boldsymbol{X}}_{\text{pl}}^{\text{mis}}$ and $\widehat{\boldsymbol{X}}_{\text{in}}^{\text{mis}}$ are sets of imputed values for unknown planting and inspection dates; and $\boldsymbol{\mathcal{T}}$ is the set of inspection type data (inspection on flowers or leaves). Thus, Eq (S18) provides the full form of the posterior distribution written in a simplified form in Eq (S1).

A Gibbs sampler is used to sequentially (in random order) sample subsets of parameters using the following set of conditional distributions:

$$f(\boldsymbol{Z} \,|\, \mu, \sigma, \beta, \theta_1, \theta_2, \rho, \boldsymbol{p}, \widehat{\boldsymbol{X}}_{\text{pl}}^{\text{mis}}, \widehat{\boldsymbol{X}}_{\text{in}}^{\text{mis}}, \boldsymbol{Y}, \boldsymbol{X}_{\text{pl}}, \boldsymbol{X}_{\text{in}}, \kappa), \tag{S19}$$

$$f(\widehat{\boldsymbol{X}}_{\text{pl}}^{\text{mis}} \,|\, \mu, \sigma, \beta, \theta_1, \theta_2, \rho, \boldsymbol{p}, \boldsymbol{Z}, \widehat{\boldsymbol{X}}_{\text{in}}^{\text{mis}}, \boldsymbol{Y}, \boldsymbol{X}_{\text{pl}}, \boldsymbol{X}_{\text{in}}, \kappa), \tag{S20}$$

$$f(\widehat{\boldsymbol{X}}_{\text{in}}^{\text{mis}} \,|\, \mu, \sigma, \beta, \theta_1, \theta_2, \rho, \boldsymbol{p}, \boldsymbol{Z}, \widehat{\boldsymbol{X}}_{\text{pl}}^{\text{mis}}, \boldsymbol{Y}, \boldsymbol{X}_{\text{pl}}, \boldsymbol{X}_{\text{in}}, \boldsymbol{\mathcal{T}}, \kappa), \tag{S21}$$

$$f(\mu, \sigma, \beta, \theta_1, \theta_2, \rho, \boldsymbol{p}, \,|\, \boldsymbol{Z}, \widehat{\boldsymbol{X}}_{\text{pl}}^{\text{mis}}, \widehat{\boldsymbol{X}}_{\text{in}}^{\text{mis}}, \boldsymbol{Y}, \boldsymbol{X}_{\text{pl}}, \boldsymbol{X}_{\text{in}}, \kappa). \tag{S22}$$

To ensure initialisation of the MCMC with a classification $\boldsymbol{Z}$ generating a finite log-likelihood, each chain is initialised with a number of introduction patches κ' much larger than the imposed number κ. The extra introduction patches are removed using greedy Metropolis-Hastings steps until κ'=κ (in practice, this takes just a few hundreds of iterations). Thereafter, the conditional distribution for the set $\boldsymbol{Z}$ (Eq S19) is sampled using block proposals (that maintain a constant κ) of various sizes in a Metropolis-Hastings sampler. Data augmentation steps (Eqs S20 and S21) are performed using block Metropolis-Hastings samplers, where block proposals of various sizes are drawn from the empirical distribution associated with each missing data component.

The distribution depicted in Eq (S22) is sampled as follows. Let $\widetilde{\Theta}$ represent a transformation of the parameter block $\Theta=\{\mu, \sigma, \beta, \theta_1, \theta_2, \rho, \boldsymbol{p}\}$ that enables to sample these parameters on an unbounded parameter space. In $\widetilde{\Theta}$, parameters ρ and p_i that are defined on (0,1) are sampled on the logit scale, and parameters that are not defined below zero ($\mu, \sigma, \theta_1, \theta_2, \beta$) are sampled on the logarithmic scale. Where appropriate, standard change-of-variable corrections are used to transform prior distributions to their associated sampling scales. The transformed parameter block is sampled using a Metropolis-Hastings algorithm [2] with multivariate Gaussian proposal distribution and adaptive covariance matrix. The details of this adaptive scheme are given below.

Various adaptive Metropolis-Hastings algorithms have been proposed [3–5]. We use a standard approach with proposals $\widetilde{\Theta}_t^*$ generated from:

$$\widetilde{\Theta}_t^* \sim \mathcal{N}\Big(\widetilde{\Theta}_{t-1}, \frac{2.38^2}{\dim(\widetilde{\Theta}_{t-1})}\widehat{\Sigma}_{t-1}\Big), \tag{S23}$$

where $\widehat{\Sigma}_{t-1}$ is an estimate of covariance in the posterior samples of $\widetilde{\Theta}$. This scheme is known to provide optimal mixing in simple theoretical examples [6]. Estimates of the covariance in Eq (S23) are updated during adaptive burn-in using the following iterative procedure:

$$\widehat{E}[\widetilde{\Theta}_{t+1}] = \frac{(\mathcal{C}_t - 1)\widehat{E}[\widetilde{\Theta}_t] + \widetilde{\Theta}_t}{\mathcal{C}_t} \tag{S24}$$

$$\Delta_t = \widetilde{\Theta}_t - \widehat{E}[\widetilde{\Theta}_{t+1}]$$

$$\widehat{\Sigma}_{t+1} = \frac{\Delta_t \otimes \Delta_t}{\mathcal{C}_t} + \frac{\mathcal{C}_t - 2}{\mathcal{C}_t - 1}\widehat{\Sigma}_t \tag{S25}$$

where $\widehat{E}[\widetilde{\Theta}_{t+1}]$ is an estimate of the sample mean at iteration t+1, $\otimes$ is the outer product, and $\mathcal{C}_t$ is a counter. In many adaptive Metropolis-Hastings samplers, $\mathcal{C}_t$ is simply set to the number of iterations of the MCMC.

However, such schemes can become prematurely inflexible, and this can lead to suboptimal mixing. To avoid this problem, we use a counter that only increases when the sampler accepts a proposal and decreases each time the sampler encounters a new area of parameter space that provides a non-negligible increase in the maximum posterior log-likelihood estimate. Thus, we define counter $\mathcal{C}_t$ as follows:

$$L'_t = \max(L_t, L'_{t-1}) \tag{S26}$$
$$\mathcal{Z}_t = \mathcal{Z}_{t-1}\ \exp(L'_{t-1} - L'_t) + \exp(L_t - L'_t) \tag{S27}$$
$$\mathcal{W}_t = \exp(L_t - L'_t)/\mathcal{Z}_t \tag{S28}$$
$$\mathcal{C}_t = \max\Big(\mathcal{C}_{\min}, \mathbb{1}_{\text{accept}(\widetilde{\Theta}^*_t)} + (1 - \mathcal{W}_t) \times \mathcal{C}_{t-1}\Big), \tag{S29}$$

where L_t is the log-likelihood at the end of iteration t and L'_t indicates the maximal log-likelihood encountered since the first iteration. When a large gain in L' is encountered, $\mathcal{W}_t{\approx}1$; this effectively resets $\mathcal{C}_t$ to some specified minimal value $\mathcal{C}_{\min}$ (we use $\mathcal{C}_{\min}{=}250$). Conversely, when no (or only negligible) augmentation of L' has been encountered for a long time, $\mathcal{Z}_t$ is free to grow (we use $\mathcal{Z}_0{=}1$), the weights $\mathcal{W}_t$ become small, and $\mathcal{C}_t$ increases by approximately one unit each time the sampler (Eq S22) accepts a proposal. Clearly, once the MCMC has generated a sample very close to the maximum posterior log-likelihood, $\mathcal{C}_t$ starts to grow monotonically and then the adaptation scheme becomes less and less flexible. Adaptation of the covariance in Eq (S25) is used during a burn-in period that is stopped once $\mathcal{C}_t{>}\mathcal{C}_{\text{Target}}{+}\mathcal{C}_{\min}$. Adaptive burn-in is turned back on if either i) further sampling generates large log-likelihood gains that reduce $\mathcal{C}_t$ sufficiently to violate this inequality, or ii) the acceptance rate becomes less than 1/200. In case (ii), we reset $\mathcal{C}_t$ to $\mathcal{C}_{\min}$. We set $\mathcal{C}_{\text{Target}}{=}10000$ for the simulation studies and $\mathcal{C}_{\text{Target}}{=}20000$ for the analysis of the real epidemic.

Two constraints are employed to prevent convergence to degenerate solutions in early MCMC iterations: i) any Metropolis-Hastings proposal giving a mean latent period greater than 10 years is rejected; ii) Metropolis-Hastings proposals are rejected if the expected prevalence is greater than 30% following an uncontrolled 4-year epidemic initialised with a single infectious individual in a landscape comprising just one patch. Constraint (ii) involves simulating an SEH sub-model in the patch containing the median number of planted trees. Since the areas of parameter space banned by these constraints are extremely unrealistic for our biological model, any bias associated with these truncations is assumed to be negligible. Indeed, analysis of MCMC output indicates that posterior distributions are located far from the bounds imposed on the parameter space.

In the simulation studies, 10 chains were run for 25000 post burn-in iterations for each simulated epidemic. To analyse the real epidemic, 30 chains were run for 10^5 post burn-in iterations for each value of κ. In both cases, the sampled parameters were stored every 25 iterations. Unless stated otherwise, all results are based on the chain with the highest mean posterior likelihood. Simulation and estimation algorithms are written in C and called from R. MCMC convergence diagnostics are performed using the R package *coda* [7]. Calculation of Eq (6) of the main text is performed using the DCUTRI algorithm of the software CaliFloPP [8].

Text D: Model Selection for K

To identify the number of introduction patches κ, we use the Fisher information of the sample $\mathcal{I}(\kappa)$, which is inversely proportional to the variance of the estimator of κ. This relationship implies that low-variance estimators can be found at the point of greatest curvature in the log-likelihood of the observed data. Thus, we seek the value of κ that maximises the expectation:

$$\mathcal{I}(\kappa) = E\left[-\Delta^2(L_{Y;\kappa})|\Theta\right] \tag{S30}$$
$$= \int -\Delta^2(L_{y;\kappa})l(y;\kappa)dy \tag{S31}$$
$$\approx -\frac{1}{C}\sum_{c=1}^{C}\Big(L_{\widehat{Y}_c;\kappa+1} - 2L_{\widehat{Y}_c;\kappa} + L_{\widehat{Y}_c;\kappa-1}\Big) \tag{S32}$$
$$\approx -\bar{L}_{Y,\kappa+1} + 2\bar{L}_{Y,\kappa} - \bar{L}_{Y,\kappa-1}, \tag{S33}$$

where Δ^2 is the centred second difference operator and $L_{Y;\kappa}{=}\ln\big[l(Y|\Theta,\kappa)\big]$ is the logarithm of the observed data likelihood. One possible approximation of the integral (Eq S31) is to use Monte Carlo approximation (Eq

S32) where $\widehat{Y_c}$ are replicated datasets generated during the MCMC runs for κ+1, κ and κ-1. An alternative approximation (Eq S33) is the second difference in the mean log-likelihoods of the observed data generated by the MCMC, given κ. We use this second approach since it has the advantage over Eq (S32) that no computation time is spent performing additional simulations. Approaches of this kind lead to estimators that are robust against over-parametrisation [9].

References

1. Gourieroux C, Monfort A, Trognon A. Pseudo Maximum Likelihood Methods: Theory. Econometrica. 1984;52(3):681–700.
2. Hastings WK. Monte Carlo sampling methods using Markov chains and their applications. Biometrika. 1970;57(1):97–109.
3. Haario H, Saksman E, Tamminen J. An adaptive Metropolis algorithm. Bernoulli. 2001;7(2):223–242.
4. Vihola M. Robust adaptive Metropolis algorithm with coerced acceptance rate. Stat Comput. 2012;22(5):997–1008.
5. Griffin JE, Walker SG. On adaptive Metropolis-Hastings methods. Stat Comput. 2013;23(1):123–134.
6. Gelman A, Carlin JB, Stern HS, Rubin DB. Bayesian Data Analysis. 2nd ed. Texts in Statistical Science Series. Chapman & Hall/CRC; 2004.
7. Plummer M, Best N, Cowles K, Vines K. CODA: Convergence diagnosis and output analysis for MCMC. R News. 2006;6(1):7–11.
8. Bouvier A, Kiêu K, Adamczyk K, Monod H. Computation of the integrated flow of particles between polygons. Environ Model Softw. 2009;24(7):843–849.
9. Evanno G, Regnaut S, Goudet J. Detecting the number of clusters of individuals using the software STRUCTURE: a simulation study. Mol Ecol. 2005;14(8):2611–2620.
